# Prepandemic levels of cytokines and immunoglobulins and risk of SARS-CoV-2 infection and COVID-19 in the general population of Barcelona

**DOI:** 10.3389/fpubh.2025.1548456

**Published:** 2025-08-20

**Authors:** Miquel Porta, José Pumarega, Ruth Aguilar, David Prieto-Merino, Laura Campi, Cristina Rius, Judit Villar-García, Marta Vidal, Alfons Jimenez, Antonio Peña, Miguel-Ángel Muñoz, Leonardo Trasande, Francisco Bolúmar, Gemma Moncunill, Magda Gasull, Carlota Dobaño

**Affiliations:** ^1^School of Medicine, Universitat Autònoma de Barcelona, Barcelona, Spain; ^2^Hospital del Mar Research Institute (IMIM), Barcelona, Spain; ^3^CIBER de Epidemiología y Salud Pública (CIBERESP), Barcelona, Spain; ^4^Division of Environmental Pediatrics, School of Medicine, New York University, New York, NY, United States; ^5^Department of Epidemiology, Gillings School of Global Public Health, University of North Carolina at Chapel Hill, Chapel Hill, NC, United States; ^6^Universitat Pompeu Fabra, Barcelona, Spain; ^7^ISGlobal, Barcelona, Spain; ^8^Facultat de Medicina i Ciències de la Salut, Universitat de Barcelona (UB), Barcelona, Spain; ^9^University of Alcalá de Henares, Madrid, Spain; ^10^Agència de Salut Pública de Barcelona, Barcelona, Spain; ^11^CIBER de Enfermedades Infecciosas (CIBERINFEC), Barcelona, Spain; ^12^Institut Universitari per a la Recerca a l’Atenció Primària de Salut Jordi Gol’, Catalan Institute of Health, Barcelona, Spain; ^13^Department of Population Health, New York University, New York, NY, United States; ^14^New York University Wagner School of Public Service, New York, NY, United States; ^15^City University of New York, New York, NY, United States

**Keywords:** cytokines, immunoglobulins, etiology, risk factors, SARS-CoV-2, COVID-19, mixtures

## Abstract

**Background:**

From a public health perspective it is remarkable that there are yet no longitudinal studies in the general population investigating the influence of the basal immune state, measured before the pandemic, on the risk of SARS-CoV-2 infection and COVID-19.

**Objective:**

To investigate the specific and combined effects of personal levels of cytokines and immunoglobulins—measured in individuals’ blood 4 years before the pandemic—on the risk of SARS-CoV-2 infection and COVID-19 in a general population.

**Methods:**

We conducted a prospective cohort study in 240 individuals from the general population of Barcelona. Thirty cytokines and 31 immunoglobulins were quantified in prepandemic serum samples (collected in 2016–17) by high-throughput multiplex quantitative suspension array technology.

**Results:**

Higher concentrations in 2016–17 of IL-8 and TNF-*α* significantly decreased the risk of SARS-CoV-2 seropositivity in 2020–21, whereas higher concentrations of MIP-1α were a risk factor for seropositivity. Most cytokines in mixtures with IL-8, MIP-1α, TNF-α or G-CSF were associated with SARS-CoV-2 seropositivity (all OR ≥2.0 or OR≤0.4 and *p* < 0.05). The five individual isotype-antigen pairs more clearly associated with seropositivity were: protectively, IgG to CMV pp150, IgG to CMV pp65, and IgG to N OC43; and, increasing risk of seropositivity, IgM to CMV pp65 and IgM to EBV EA-D. The four cytokines most consistently associated with the risk of COVID-19 were also G-CSF, IL-8, TNF-*α*, and MIP-1α. The four isotype-antigen pairs more strongly associated with risk of COVID-19 (all protective) were IgA to CMV pp65 and N 229E, and IgG to EBV EAD and VCAp18.

**Conclusion:**

The unique longitudinal design of this study, with measurements before and during the pandemic in a general population, provides novel knowledge on the protective and detrimental effects of specific individual cytokines and immunoglobulins, and their mixtures, on the risk of SARS-CoV-2 seropositivity and COVID-19. If confirmed, findings would be significantly relevant for medicine and public health.

## 1 Introduction

The basal immune state represents the baseline level of immune activity and preparedness against an infeccion or other immune stimuli, and encompasses the innate and acquired immune systems. The innate immune system acts as a general first line of defense against pathogens, while the acquired immune sytem develops specific response to pathogens, both playing a pivotal role in determining the body’s response to infections. Interrelated with each person’s characteristics, health status, past exposures, lifestyle, and living conditions, the basal immune state is a key factor to help explain a phenomenon that was evident during the COVID-19 pandemic, and which remains partly unexplained: the wide heterogeneity in immunological and clinical responses to SARS-CoV-2 infection (1–8). Today, for instance, the capacity of pre-existing immunity to human common coronaviruses (HCoV) to crossprotect against *de novo* COVID-19 is still largely unknown.

Investigating the impact of the basal immune state on the susceptibility to SARS-CoV-2 is crucial to advance our understanding of COVID-19 dynamics and to improve outcomes. In spite of these evidences, there are yet no longitudinal studies investigating the influence of the basal immune state measured before the pandemic on the risk of SARS-CoV-2 infection (defined by a positive rRT-PCR or seropositivity to one or several viral antigens) and development of COVID-19 (symtomatology due to the infection): thus far, virtually all studies on levels of cytokines and immunoglobulins, and SARS-CoV-2 infection and COVID-19 have been conducted with biological samples collected during the pandemic, in individuals—likely exposed to SARS-CoV-2, infected, or ill, often severely—who sought medical attention in health facilities. Hence, such studies could only assess the role of cytokines and immunoglobulins as markers of disease severity and prognosis, not as co-etiologic factors (6, 9, 10). To ensure a proper time sequence, assessing the possible influence of basal cytokine and immunoglobulin levels on the risk of SARS-CoV-2 infection and COVID-19 requires that such biomarkers were measured before the pandemic outbreak.

Therefore, the present study aimed to investigate the specific and combined effects of personal levels of cytokines and immunoglobulins—measured in individuals’ blood 4 years before the pandemic—on the risk of SARS-CoV-2 infection and COVID-19 in the general population of Barcelona.

## 2 Methods

### 2.1 Study population

The present prospective cohort study was based on the Barcelona Health Survey (BHS) of 2016, whose methods have been described in detail (6, 7, 11, 12). The BHS generated a sample representative of the general, adult, non-institutionalized population of the city of Barcelona (Spain). Through face-to-face interviews, the survey collected information about sociodemographic factors, chronic disorders, life styles, uses of healthcare services and preventive practices. At the end of the 2016 BHS interview, participants were offered to take part in a health examination, and 240 individuals accepted. Subsequently, between July 2016 and May 2017, a nurse interviewed again face-to-face such individuals, measured body parameters, and collected blood and urine samples (6, 11). Participants had been asked to fast for at least 8 h before blood extraction. Blood was collected in a vacuum system tube and centrifuged for 15 min x 3000 rpm at 4°C to obtain serum, which was divided in 1–3 mL aliquots and stored at −80°C (6, 11). The prepandemic levels of the cytokines and immunoglobulins assessed in the present report were analyzed in such serum samples (see sections 2.3., 2.4., and 2.5. below).

After scientific, financial and logistic preparations, the 240 participants began to be invited to a follow-up visit in October 2020, in a severe phase of the pandemic, and 174 (72.5%) attended between November 2020 and June 2021 (6). Thus, for the present analyses our study spans from 2016 to 17, when the baseline interviews and collection of biological samples first took place, to 2020–21, when the follow-up visit and collection of biological samples took place again. During the follow-up visit a nurse measured their weight, height. She also collected a nasopharyngeal swap, and new blood and urine samples, which constitute a crucial scientific resource of the present cohort study to analyze immunological components of the SARS-CoV-2 infection. The median time between the extraction of biological samples in 2016–17 and 2020–21 was 4.1 years. Compared to the 66 subjects who did not attend the follow-up visit, the 174 participants were more commonly women, younger, born in Catalonia, with a lower body mass index (BMI), more affluent, and with better self-perceived health (6). The main analyses reported in the present paper are based on 154 individuals (72 men, 82 women) who had not received any COVID-19 vaccine at the time of the follow-up visit (i.e, excluding 20 participants who had received a COVID-19 vaccine). Characteristics of participants have been published in Table 1 of Ref. (6).

The Ethics Committee of the Parc de Salut Mar reviewed and approved the study protocols, and all participants signed an informed consent before sample collection and completing questionnaires (11). All methods were performed in accordance with the relevant guidelines and regulations.

### 2.2 Socioeconomic and living conditions

Shortly before the follow-up visit in 2020–2021, the participants completed an online survey concerning signs and symptoms of COVID-19, diagnostic tests performed and their results, use of healthcare services, and vaccination, all during the previous months of the pandemic. This information was ascertained as well with the data base of the System of Diseases of Mandatory Reporting of the Agency of Public Health of Barcelona, and of the Public Data Analysis for Health Research and Innovation Program of Catalonia (PADRIS) of the Catalan Agency for Health Quality and Evaluation (AQUAS). The PADRIS databases contain detailed records on demographics, diagnoses of all medical conditions and comorbidities, laboratory results, medications dispensed by pharmacies, visits to Primary Care physician, procedures, and medical admissions from public hospitals for the whole population of Catalonia. This data was used to complement information collected during the study (8). During follow-up the study also collected information on participants’ lifestyle and living conditions during the pandemic. During the visit, the nurse clarified answers to the online survey and asked further questions on vaccination, weight changes, and pregnancies. A household outdoor index was computed taking into account the number of individuals living in the same household, the availability and use of an outdoor space. Other factors included in the online survey were: work conditions, use of public and private transport, and individual measures taken to avoid infection (6, 7).

### 2.3 Quantification of cytokines, chemokines and growth factors

The Cytokine Human Magnetic 30-Plex Panel from Invitrogen™ was used to measure concentrations (pg/mL) of the following 30 cytokines, chemokines and growth factors in serum samples collected in 2016–17 (thus, prepandemic) (8, 13, 14): epidermal growth factor (EGF), fibroblast growth factor (FGF), granulocyte colony-stimulating factor (G-CSF), granulocyte-macrophage colony-stimulating factor (GM-CSF), hepatocyte growth factor (HGF), vascular endothelial growth factor (VEGF), tumor necrosis factor (TNF), interferon (IFN)-*α*, IFN-*γ*, interleukin (IL)-1RA, IL-1β, IL-2, IL-2R, IL-4, IL-5, IL-6, IL-7, IL-8, IL-10, IL-12(p40/p70), IL-13, IL-15, IL-17, IFN-γ induced protein (IP-10), monocyte chemoattractant protein (MCP-1), monokine induced by IFN-γ (MIG), macrophage inflammatory protein (MIP)-1α, MIP-1β, regulated on activation normal T cell expressed and secreted (RANTES) and eotaxin. Each assay plate included 16 serial dilutions (2-fold) of a standard curve, and two blank controls. Samples were acquired on a Luminex 100/200 instrument and analyzed in xPONENT software 3.1. The concentration of each analyte was obtained by interpolating the median fluorescent intensity (MFI) to a 5-parameter logistic regression curve and reported as pg./mL using the drLumi R package. Limits of quantification (LOQ) were estimated based on cutoff values of the 30% coefficient of variation (CV) of the standard curve for each analyte (13). When the value of an analyte was below the lower LOQ (lLOQ), the mid-value of this limit for the corresponding laboratory plate was assigned; and when a value was above the corresponding upper LOQ (uLOQ), the assigned value was twice this uLOQ. Limits of quantification, percentages of quantification, and concentrations obtained for each cytokine have been published in Supplementary Table 1 and Table 1 of Ref. (8).

### 2.4 Serology of viral exposures

The levels of IgM, IgA and IgG against the Nucleocapsid (N) protein of the 4 human common cold coronavirus (HCoV-229E, OC43, NL63, HKU1), two Epstein–Barr virus (EBV) antigens (EA-D, VCA p18), and two Cytomegalovirus (CMV) antigens (pp65, pp150), were assessed by high-throughput multiplex quantitative suspension array technology (qSAT) in a FlexMap3D instrument as previously described, and data QA/QC and preprocessing were performed with R (8, 15). Briefly, antigen-coupled beads were added to a 384-well μClear^®^ flat bottom plate in multiplex. A hyper-immune plasma pool at 3-fold 10 serial dilutions starting from 1:250 was used as positive control in each assay plate for QA/QC and calibration purposes. Final dilution of test samples was 1:500. To quantify IgA and IgM, samples and controls were pre-treated with anti-human IgG (Gullsorb) at 1:10 dilution, to avoid IgG interferences. MFI was reported for each isotype-antigen pair. Levels of each immunoglobulin have been published in Table 4 of Ref. (8).

### 2.5 Quantification of total immunoglobulins

The quantification of total immunoglobulins (IgE, IgA, IgM, IgG1, IgG2, IgG3, and IgG4) was performed with the Antibody Isotyping 7-Plex Human ProcartaPlex™ panel (Thermo Fisher Scientific, Vienna, Austria) following the manufacturer’s instructions. Samples were tested at a dilution of 1/200000 and a second dilution of 1/500000, acquired on a Luminex 100/200 instrument and analyzed in xPONENT software 3.1. The concentration of each isotype was obtained by interpolating the MFI to a 5-parameter logistic regression curve and reported as μg/mL (8). The analyses of the present report use the levels of total immunoglobulins obtained with the dilution of 1/200000. The IgG1 and the IgG3 were not quantified in 1.7 and 20.8% of serum samples, respectively, and imputations of the missing values were based on the levels obtained by the second dilution. We computed the arithmetic sum of levels of the four total IgG subclasses (IgG1, IgG2, IgG3, and IgG4) (8).

Intraindividual changes in cytokines and immunoglobulins between 2016–17 and 2020–21 were moderate, and similar between participants who in 2020–21 were SARS-CoV-2 seropositive and seronegative, and between participants who did and did not develop COVID-19 (8). The similarity suggests that it is valid to use the prepandemic levels of cytokines and immunoglobulins to assess the risk relationship (protective or harmful) of these basal immune markers with the development of SARS-CoV-2 seropositivity and COVID-19, which is the main object of the present paper.

### 2.6 Determination of SARS-CoV-2 infection and COVID-19

#### 2.6.1 SARS-CoV-2 infection

SARS-CoV-2 infection was determined at the Center for Genomic Regulation (CRG) in all 174 members of the cohort who attended the follow-up visit in 2020–2021 by real time reverse-transcriptase polymerase chain reaction (rRT-PCR) in nasopharyngeal swabs. Briefly, samples were collected in 600 μL of lysis solution (DNA/RNA Shield, Zymo) to inactivate the virus, break membranes and stabilize the RNA. Samples were processed in a TECAN Dreamprep robot to isolate the RNA using the Quick-DNA/RNA Viral MagBead kit (Zymo; #R2140), and the purified RNA was analyzed by rRT-PCR in a ABI 7900 HT (384 wells) following the CDC standard procedure. Positive and negative controls were included in each assay plate. Among the 174 participants, there were 4 rRT-PCR-positives (6).

To detect previous SARS-CoV-2 infections, antibody serological status of each participant was assessed in serum samples analyzed at the ISGlobal Immunology Laboratory in Barcelona. The MFI levels of IgG, IgM and IgA against 5 SARS-CoV-2 antigens were assessed by high-throughput multiplex qSAT (5, 6, 16), as described in section 2.4 for the other viral exposures. The five antigens from SARS-CoV-2 were the Spike (S) protein and the Receptor Binding Domain (RBD; both fused with C-terminal 6xHis and StrepTag purification sequences and purified from supernatant of lentiviraltransduced CHO–S cells cultured under a fed-batch system), the S1 (aa1–681, expressed in Expi293 and His tag-purified), the S2 subunit (purchased from SinoBiologicals), the Nucleocapsid full length protein (NFL), and its C-terminal (NCt; expressed in *E. coli* and His tagpurified) (6, 7, 16).

Of the 154 participants mentioned above, 41 were SARS-CoV-2 seropositive (26.6%) at the time of the follow-up visit in 2020–21 (including all 4 positives by the follow-up rRT-PCR), 9 indeterminate (5.8%), and 104 seronegative (67.5%). There were no major differences in the main characteristics of seropositive and seronegative participants [Supplementary Table 5 of Ref. (6)].

#### 2.6.2 COVID-19

Cases of COVID-19 have been described in detail (6, 7). In total there were 20 cases of COVID-19 at the time of the follow-up visit in 2020–21. All were seropositive for SARS-CoV-2 in our immunological assay, all reported COVID-19 related symptoms, and 2 of them had been hospitalized. Specifically, 10 cases provided information of a positive diagnostic test for SARS-CoV-2 infection (including all 4 positives at the follow-up rRT-PCR), and 2 or more COVID-19 related signs or symptoms; 2 were diagnosed of COVID-19 by a physician; and 8 had COVID-19 related signs or symptoms (6, 7, 17). There were no major differences in the main characteristics of participants with and without COVID-19 [Table 1 of Ref. (6)].

### 2.7 Statistical analyses

Univariate and multivariate analyses were performed as customary (8, 18). Levels of cytokines and immunoglobulins were initially categorized as tertiles. Cut-off points for tertiles were based on the distribution of the levels in the 240 participants [see Tables 1, 4 in Ref. (4)]. Some cytokines and immunoglobulins were also dichotomized if no linear dose–response was apparent in tertile analyses, or if cell size was small, and in the absence of substantive knowledge on a normal or natural cutpoint (6, 7). Cytokine and immunoglobulin levels were also analyzed as continuous variables base 10 log-transformed (8).

The main effects of each biomarker of interest (cytokines and immunoglobulins) were independently explored in base models including the inflammatory and immunological single-biomarker in each separate model, and potential confounders (data on the latter drawn from our online follow-up survey, personal interviews, and follow-up visit, see 2.1. and 2.2. above) (6, 7, 18). To assess the effects of mixtures of cytokines and immunoglobulins, mutually adjusted for, we built multi-biomarker regression models and selected groups of 2 to 6 biomarkers that had been significant in their single-biomarker models; we selected mixtures in which all or most elements showed associations with the outcome. The clinical and epidemiological literature on cytokines and immunoglobulins, and SARS-CoV-2 infection and COVID-19 was also considered when building these multi-biomarker models (8). For instance, because a recent report found that high serum levels of IL-6, IL-8 and TNF-*α* concentrations at the time of hospitalization were strong and independent predictors of survival in hospitalized patients with COVID-19 (9), we also built multi-biomarker models with the combinations of these three cytokines to analyze their combined effect on the risk of the SARS-CoV-2 infection and COVID-19.

**Figure 1:**
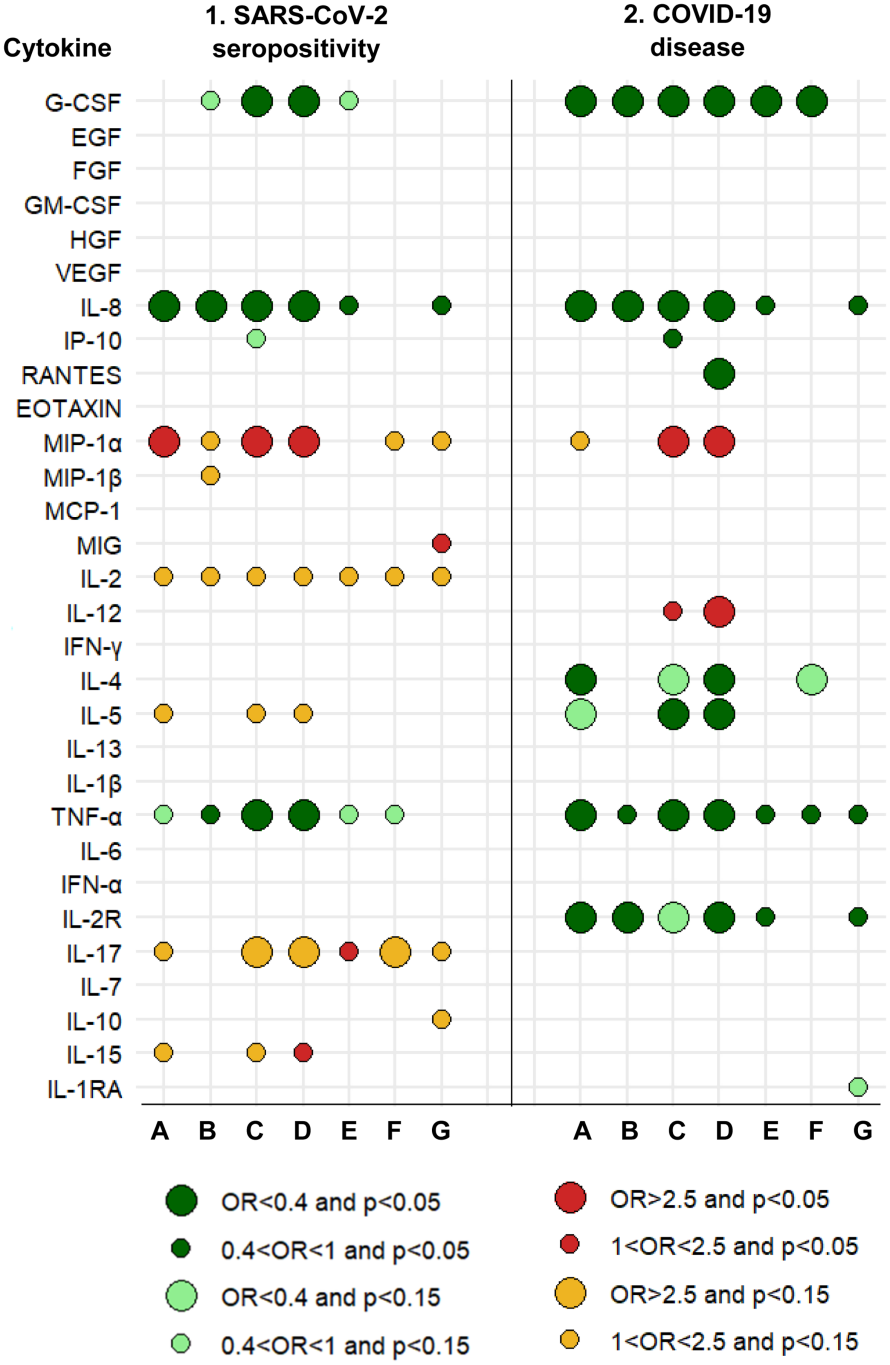
Graphical summary of results and relationships between cytokines measured in 2016–17 and the risk of SARS-CoV-2 seropositivity and COVID-19 disease in 2020–21. A protective effect (OR<1) is shown by greens. An increased risk (OR>1) is shown by reds. The size of the circle is determined by the magnitude of the OR. The intensity of the color of the circle is determined by the magnitude of the *p*-value. A: Logistic regression with each cytokine dichotomous (based on analyses whose results are summarized in Tables 1, 6). B: Logistic regression with each cytokine continuous (based on analyses whose results are summarized in Tables 1, 6). C: Logistic regression, mixture of cytokines (circles are based on all multivariate models, a selection of which is shown in Tables 2, 7). D: Logistic regression, mixture of cytokines and immunoglobulins (circles are based on all multivariate models, a selection of which is shown in Tables 5, 10). E: Linear regression with each cytokine continuous. F: Linear regression for censored data. G: Quasi-binomial generalized linear regression. See Methods (2.7. Statistical analyses).

**Figure 2:**
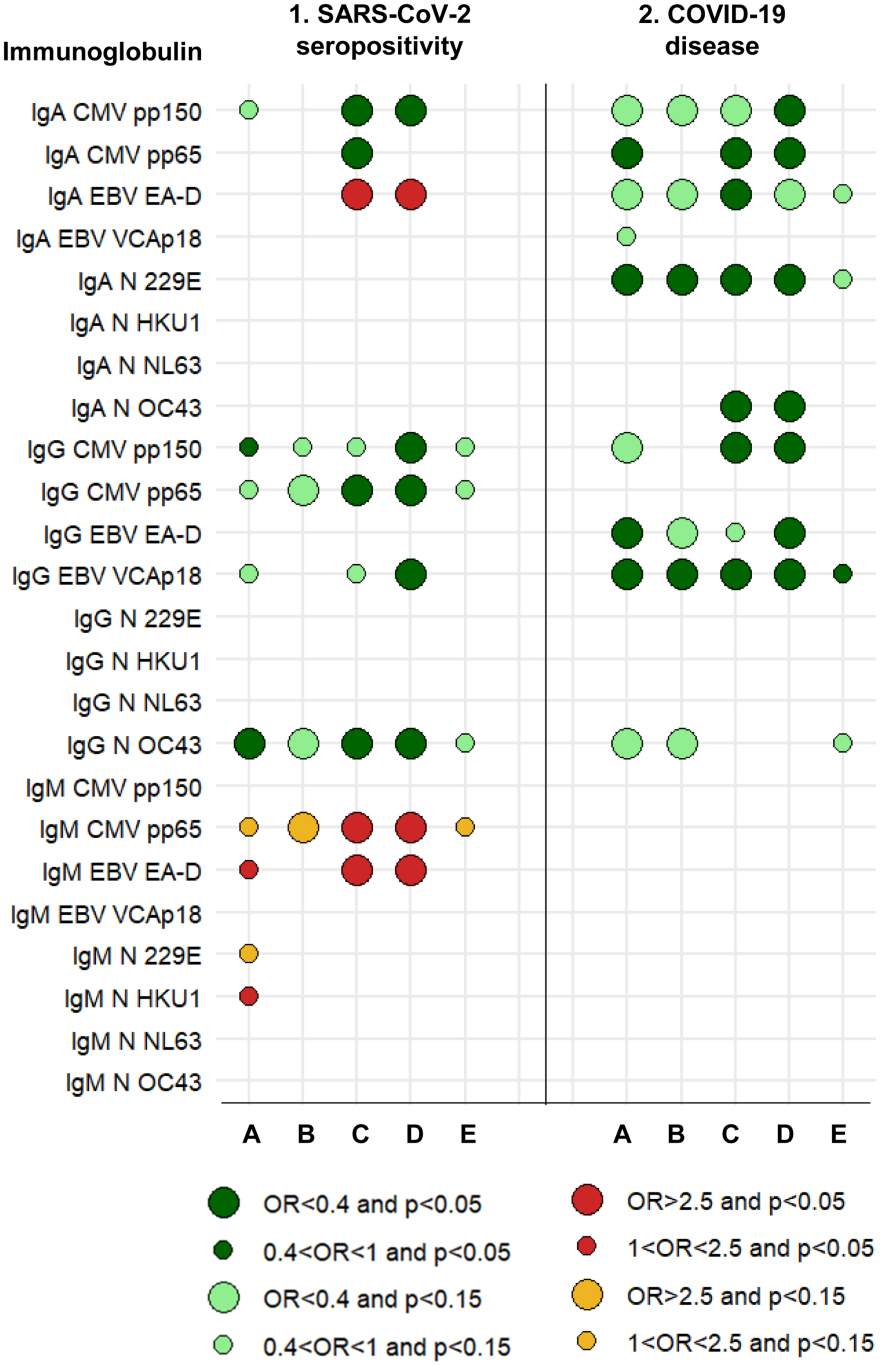
Graphical summary of results and relationships between immunoglobulins measured in 2016–17 and the risk of SARS-CoV-2 seropositivity and COVID-19 disease in 2020–21. A protective effect (OR<1) is shown by greens. An increased risk (OR>1) is shown by reds. The size of the circle is determined by the magnitude of the OR. The intensity of the color of the circle is determined by the magnitude of the *p*-value. A: Logistic regression with each immunoglobulin dichotomous (based on analyses whose results are summarized in Tables 3, 8). B: Logistic regression with each immunoglobulin continuous (based on analyses whose results are summarized in Tables 3, 8). C: Logistic regression, mixture of immunoglobulins (circles are based on all multivariate models, a selection of which is shown in Tables 4, 9). D: Logistic regression, mixture of immunoglobulins and cytokines (circles are based on all multivariate models, a selection of which is shown in Tables 5, 10). E: Linear regression with each immunoglobulin continuous. See Methods (2.7. Statistical analyses).

To assess the magnitude of the associations, odds ratios (OR) between levels of the biomarkers of inflammation and of immunological status, and the two outcomes (SARS-CoV-2 seropositivity and COVID-19), with their corresponding 95% confidence intervals (CI) were computed through unconditional logistic regression (18). For the SARS-CoV-2 analyses, the 9 participants with indeterminated SARS-CoV-2 seropositivity were excluded. ORs were adjusted for age, sex, tobacco smoking, BMI, education, the household outdoor index or other socioeconomic variables if such potentially confounding variables fulfilled pre-established criteria: *p* ≤ 0.5 to enter the model and *p* ≤ 0.25 to remain in it in a stepwise procedure. To assess significance, we considered the magnitude of the association (e.g., OR ≥2.0 or OR≤0.4), the precision of the effect estimate, and the statistical significance (e.g., *p* < 0.05 or *p* < 0.15) (6, 7, 18, 19). While in tables we provide a wide spectrum of positive and negative results (i.e., suggesting potential associations as well as lack of association, as in Table 1), in Figures 1, 2 we represent a summary of findings (increasing or decreasing risks, blanks showing no associations), and in the main text of the Results section we focus only on the most significant associations based on the criteria just mentioned.

**Table 1:** Effect of individual cytokine levels measured in 2016–17 on the risk of SARS-CoV-2 seropositivity in 2020–21 (*N* = 145)*.

Cytokine	OR^a^	(95% CI)	*p* ^b^
Growth factors			
G-CSF			
T1	1.00		0.515
T2	0.59	(0.24−1.48)	
T3	0.73	(0.30−1.76)	
T1	1.00		0.278
T2 + T3	0.66	(0.31−1.40)	
Continuous^c^	0.65	(0.39−1.09)	0.104
EGF^d^			
T1	1.00		0.595
T2	1.25	(0.49−3.24)	0.309^e^
T3	1.62	(0.64−4.13)	
Continuous^c^	1.58	(0.80−3.11)	0.187
FGF			
T1	1.00		0.651
T2	0.65	(0.26−1.65)	
T3	0.90	(0.37−2.15)	
GM-CSF			
T1	1.00		0.860
T2	1.27	(0.52−3.07)	
T3	1.21	(0.48−3.02)	
T1	1.00		0.590
T2 + T3	1.24	(0.57−2.70)	
HGF			
T1	1.00		0.270
T2	2.11	(0.85−5.23)	
T3	1.43	(0.54−3.80)	
VEGF			
T1	1.00		0.589
T2	1.51	(0.62−3.69)	
T3	1.01	(0.39−2.61)	
Chemokines			
IL-8^d^			
T1	1.00		**0.028**
T2	1.43	(0.59−3.45)	
T3	**0.36**	(0.13−0.99)	
T1 + T2	1.00		**0.011**
T3	**0.30**	(0.12−0.76)	
Continuous^c^	**0.17**	(0.04−0.75)	**0.019**
IP-10			
T1	1.00		0.564
T2	1.15	(0.49−2.68)	
T3	0.69	(0.26−1.80)	
RANTES^d^			
T1	1.00		0.390
T2	0.54	(0.19−1.51)	
T3	0.63	(0.27−1.48)	
T1	1.00		0.178
T2 + T3	0.59	(0.28−1.27)	
EOTAXIN			
T1	1.00		0.805
T2	1.37	(0.54−3.47)	
T3	1.19	(0.49−2.91)	
MIP-1α^d^			
T1	1.00		0.069
T2	0.96	(0.35−2.61)	
T3	2.46	(0.99−6.15)	
T1 + T2	1.00		**0.021**
T3	**2.52**	(1.15−5.50)	
Continuous^c^	1.61	(0.96−2.71)	0.071
MIP-1β			
T1	1.00		0.380
T2	1.43	(0.56−3.63)	0.164^e^
T3	1.95	(0.76−4.99)	
T1	1.00		0.234
T2 + T3	1.66	(0.72−3.80)	
Continuous^c^	1.69	(0.90−3.19)	0.102
MCP-1^d^			
T1	1.00		0.107
T2	0.40	(0.15−1.10)	
T3	1.16	(0.48−2.78)	
Continuous^c^	2.22	(0.47−10.62)	0.316
MIG^d^			
Not quantified	1.00		0.520
Quantified	1.29	(0.60−2.78)	
TH1			
IL-2^d^			
T1	1.00		0.286
T2	1.06	(0.41−2.74)	0.160^e^
T3	1.95	(0.76−5.03)	
T1 + T2	1.00		0.114
T3	1.90	(0.86−4.19)	
Continuous^c^	1.33	(0.91−1.94)	0.139
IL-12^d^			
T1	1.00		0.730
T2	1.01	(0.40−2.56)	0.387^e^
T3	1.38	(0.55−3.49)	
IFN-γ^d^			
Not quantified	1.00		0.458
Quantified	1.47	(0.53−4.10)	
TH2			
IL-4			
Not quantified	1.00		0.659
Quantified	1.20	(0.54−2.68)	
IL-5^d^			
Q1 + Q2^f^	1.00		0.245
Q3	1.27	(0.49−3.28)	0.103^e^
Q4	2.22	(0.87−5.65)	
≤Q3	1.00		0.107
Q4	2.06	(0.86−4.97)	
Continuous^c^	1.47	(0.66−3.28)	0.351
IL-13^d^			
T1	1.00		0.673
T2	1.35	(0.52−3.48)	0.367^e^
T3	1.50	(0.60−3.74)	
Continuous^c^	1.25	(0.74−2.11)	0.411
Pro-inflammatory			
IL-1β^d^			
T1	1.00		0.782
T2	0.88	(0.35−2.20)	
T3	1.23	(0.49−3.05)	
TNF-α			
T1	1.00		0.183
T2	0.49	(0.20−1.20)	
T3	0.48	(0.19−1.19)	
T1	1.00		0.065
T2 + T3	0.48	(0.22−1.05)	
Continuous^c^	**0.59**	(0.35−0.98)	**0.042**
IL-6			
T1	1.00		0.948
T2	0.98	(0.39−2.45)	
T3	0.87	(0.36−2.09)	
IFN-α^d^			
T1	1.00		0.290
T2	0.95	(0.37−2.46)	
T3	1.86	(0.74−4.68)	
IL-2R			
T1	1.00		0.663
T2	0.86	(0.35−2.10)	0.368^e^
T3	0.66	(0.27−1.63)	
IL-17^d^			
Not quantified	1.00		0.115
Quantified	1.88	(0.86−4.10)	
Regulatory			
IL-7			
T1	1.00		0.722
T2	0.80	(0.32−1.99)	
T3	0.69	(0.28−1.70)	
Anti-inflammatory			
IL-10^d^			
Not quantified	1.00		0.958
Quantified	1.02	(0.48−2.18)	
IL-15^d^			
Not quantified	1.00		0.118
Quantified	1.90	(0.85−4.27)	
IL-1RA^d^			
T1	1.00		0.626
T2	0.80	(0.31−2.08)	
T3	1.27	(0.52−3.09)	
Continuous^c^	1.18	(0.48−2.90)	0.722

*The odds ratios quantify the magnitude of the associations between the exposures and SARS-CoV-2 seropositivity in the 41 SARS-CoV-2 seropositives and the 104 seronegatives. OR: Odds ratio. An OR = 1 indicates the reference category. CI: Confidence interval. T1 to T3: tertiles. Q1 to Q4: quartiles. For categorical values of cytokines, ORs shown in bold are ORs ≥2.5 or ORs ≤0.4 with *p* values <0.05. For continuous values of cytokines, ORs are shown in bold if their *p* values <0.05. Cytokines IL-5, IL-15, and IL-17 will show associations with seropositivity in multivariate mixture models. It is thus worth noting that in this table above they have odds ratios for seropositivity near 2, not statistically significantly, in their respective dichotomous forms (Q4 vs. ≤Q3 for IL-5, and quantified vs. not quantified for IL-15 and IL-17). This table and Table 6 are the only parts of the article in which all 30 cytokines appear, thus including cytokines that are not associated with the respective outcomes, SARS-CoV-2 seropositivity and COVID-19.

a Unless otherwise specified, odds ratios were adjusted for household outdoor index.

b Unless otherwise specified, p-value derived from Wald’s test.

c Odds ratio for each increase of 10 times in the level of the cytokine. We present just some examples of statistically nonsignificant continuous variables; all other continuous variables not shown in the table were statistically nonsignificant.

d Odds ratios were further adjusted for smoking.

e Multivariate analog of Mantel’s extension test for linear trend.

f The category is exclusively made up of individuals whose cytokine level was less than the respective lower limit of quantification (see Methods, section 2.3).

In addition, to complement the previous strategies of analysis, we estimated three other types of regression models: (1) a linear regression with each cytokine (in the log 10 scale) as the continuous response and the seropositivity (or COVID-19) as the main explanatory variable, including in the models the corresponding adjustment covariables; (2) to account for quantification limits, we repeated the previous linear regression analysis with regression methods for censored data using the NADA2 library in R (20); and (3) we normalized the cytokine values as a proportion between 0 and 1 of the limits of quantification (value-lLOQ) / (uLOQ–lLOQ), and compared these proportions between the two groups of SARS-CoV-2 infection (seropositive and seronegative), and of COVID-19 (with and without the disease), respectively, with a quasi-binomial generalized linear regression, including as well the corresponding adjustment covariables (21). The main results of these three complementary regression models are shown in columns E to G of Figure 1 and column E of Figure 2.

All tests were two-tailed. Statistical analyses were conducted using R, version 4.3.3 (Boston, MA, 2024), and SPSS version 22.0.0.0 (IBM SPSS Statistics, Armonk, NY, 2013).

## 3 Results

### 3.1 Effects of baseline immune markers on SARS-CoV-2 seropositivity

Higher concentrations in 2016–17 of IL-8 and TNF-*α* showed some significant associations with a decreased risk of SARS-CoV-2 seropositivity in 2020–21, whereas higher concentrations of MIP-1*α* were a risk factor for seropositivity (Table 1). Thus, for instance, participants with IL-8 levels in the upper tertile were 70% less likely to be seropositive (OR = 0.30, 95% CI: 0.12–0.76); and participants with MIP-1*α* in the upper tertile were 2.5-times more likely to be seropositive (OR = 2.52, 95% CI: 1.15–5.50). Besides these three cytokines, there was little or no evidence of an association between seropositivity and any of the other 27 cytokines individually (Figure 1, columns 1. A and 1. B).

Most cytokines in mixtures with IL-8, TNF-α, MIP-1α or G-CSF were associated with SARS-CoV-2 seropositivity (Table 2, models 1a, 1b, 2a, 3a, 3b; Figure 1, column 1. C). In most of these multi-biomarker models, IL-8, TNF-α, and G-CSF had an OR≤0.4, while MIP-1α had an OR≥2.0 (all *p* < 0.05).

**Table 2:** Influence of mixtures of cytokines on the risk of SARS-CoV-2 seropositivity (*N* = 145)*.

Model		OR^a^	(95% CI)	*p* ^b^	Model	OR^a^	(95% CI)	*p* ^b^
1a	IL-8				1b			
	T1 + T2	1.00		0.016		1.00		0.019
	T3	0.31	(0.12−0.80)			0.32	(0.12−0.83)	
	IP-10							
	T1 + T2	1.00		0.092		1.00		0.064
	T3	0.41	(0.15−1.16)			0.38	(0.13−1.06)	
	MIP-1α							
	T1 + T2	1.00		0.024		1.00		0.011
	T3	2.87	(1.15−7.19)			3.50	(1.34−9.17)	
	G-CSF							
	T1	–				1.00		0.026
	T2 + T3					0.33	(0.12−0.87)	
	TNF-α							
	T1	1.00		0.022		–		
	T2 + T3	0.35	(0.15−0.86)					
	IL-17							
	Not quantified	1.00		0.103		1.00		0.063
	Quantified	2.21	(0.85−5.71)			2.60	(0.95−7.13)	
2a^c^	IL-8				2b			
	T1 + T2	1.00		0.015		1.00		0.015
	T3	0.30	(0.12−0.79)			0.31	(0.12−0.80)	
	MIP-1α							
	T1 + T2	1.00		0.014		1.00		0.008
	T3	3.10	(1.25−7.67)			3.30	(1.37−7.95)	
	G-CSF							
	T1	1.00		0.055		1.00		0.071
	T2 + T3	0.40	(0.16−1.02)			0.45	(0.19−1.07)	
	IL-5							
	≤Q3	1.00		0.125		–		
	Q4	2.17	(0.81−5.84)					
3a	IL-8				3b			
	T1 + T2	1.00		0.015		1.00		0.012
	T3	0.31	(0.12−0.80)			0.30	(0.12−0.77)	
	MIP-1α							
	T1 + T2	–				1.00		0.011
	T3					2.93	(1.28−6.71)	
	TNF-α							
	T1	1.00		0.035		1.00		0.032
	T2 + T3	0.41	(0.18−0.94)			0.40	(0.17−0.93)	
	IL-15							
	Not quantified	1.00		0.063		–		
	Quantified	2.26	(0.96−5.34)					
4a	IP-10				4b			
	T1 + T2	1.00		0.091		1.00		0.162
	T3	0.43	(0.16−1.15)			0.51	(0.20−1.31)	
	TNF-*α*							
	T1	1.00		0.017		1.00		0.027
	T2 + T3	0.35	(0.15−0.83)			0.39	(0.17−0.90)	
	IL-17							
	Not quantified	1.00		0.061		–		
	Quantified	2.41	(0.96−6.01)					
	IL-15							
	Not quantified	1.00		0.097		1.00		0.065
	Quantified	2.18	(0.87−5.46)			2.34	(0.95−5.79)	
	IL-5							
	≤Q3	–				1.00		0.160
	Q4					1.95	(0.77−4.98)	

*The odds ratios quantify the magnitude of the associations between the cytokines and SARS-CoV-2 seropositivity in the 145 individuals, 41 SARS-CoV-2 seropositives and 104 seronegatives. An OR of 1.00 denotes the reference category. T1 to T3: tertiles. Q1 to Q4: quartiles.

a Unless otherwise specified, odds ratios of the cytokines were always mutually adjusted for, and further adjusted by household outdoor index.

b Wald’s test (two-tailed).

c Odds ratios of the cytokines were mutually adjusted for, and further adjusted by household outdoor index and smoking (all two confounders *p* < 0.25).

Generally, complementary models (Figure 1, columns 1. E to 1. G; Supplementary Table 1.1) were coherent with logistic regression models for IL-8, TNF-α, G-CSF, MIP-1α, and IL-17 (Figure 1, columns 1. A to 1. D).

The five individual isotype-antigen pairs more clearly associated with SARS-CoV-2 seropositivity were: protectively, IgG to CMV pp150, IgG to CMV pp65, and IgG to N OC43; and increasing risk of seropositivity, IgM to CMV pp65 and IgM to EBV EA-D (Table 3; Supplementary Table 2; Figure 2, columns 1. A and 1. B). Mixtures of all these five isotype-antigen pairs were also associated with seropositivity (Table 4; Figure 2, columns 1. C and 1. D). Thus, participants whose IgG N OC43 levels in 2016–17 were in the upper tertile were 70% less likely to be SARS-CoV-2 seropositive in 2020–21 (OR = 0.30; Table 4, models 1a and 1b; Figure 2, column 1. C); participants with IgG CMV pp65 levels in the upper tertiles were between 65 and 61% less likely to be seropositive (ORs between 0.35 and 0.39; Table 4, models 5a and 5b); and participants with IgM CMV pp65 in the upper tertiles were near 3-times more likely to be seropositive (Table 4, models 4a and 4b).

**Table 3:** Effect of selected individual isotype-antigen pairs for cytomegalovirus, Epstein–Barr virus, and common cold infections measured in 2016–17 on the risk of SARS-CoV-2 seropositivity in 2020–21 (*N* = 145)*.

Isotype-antigen pair	OR^a^	(95% CI)	*p* ^b^
IgA CMV pp150			
T1	1.00		0.029
T2	2.62	(1.03−6.66)	
T3	0.89	(0.32−2.48)	
T1 + T2	1.00		0.098
T3	0.50	(0.22−1.14)	
Continuous^c^	0.58	(0.18−1.88)	0.366
IgA CMV pp65^d^			
T1	1.00		0.618
T2	0.95	(0.38−2.31)	
T3	0.65	(0.25−1.65)	
T1 + T2	1.00		0.444
T3	0.73	(0.33−1.62)	
IgA EBV EA-D			
T1	1.00		0.425
T2	0.77	(0.30−2.03)	
T3	1.44	(0.61−3.38)	
T1 + T2	1.00		0.226
T3	1.60	(0.75−3.43)	
IgG CMV pp150			
T1	1.00		0.102
T2	0.51	(0.21−1.24)	**0.039** ^e^
T3	**0.37**	(0.14−0.97)	
T1	1.00		**0.039**
T2 + T3	**0.44**	(0.20−0.96)	
Continuous^c^	0.47	(0.22−1.03)	0.060
IgG CMV pp65			
T1	1.00		0.200
T2	0.48	(0.19−1.19)	
T3	0.49	(0.19−1.24)	
T1	1.00		0.073
T2 + T3	0.48	(0.22−1.07)	
Continuous^c^	0.31	(0.07−1.39)	0.125
IgG EBV VCAp18			
T1	1.00		0.251
T2	0.84	(0.35−2.04)	0.108^e^
T3	0.46	(0.18−1.17)	
T1 + T2	1.00		0.106
T3	0.50	(0.22−1.16)	
Continuous^c^	0.53	(0.20−1.39)	0.198
IgG N OC43			
T1	1.00		0.071
T2	1.62	(0.68−3.84)	
T3	0.52	(0.19−1.40)	
T1 + T2	1.00		**0.045**
T3	**0.40**	(0.17−0.98)	
Continuous^c^	0.38	(0.13−1.13)	0.082
IgM CMV pp65			
T1	1.00		0.250
T2	2.19	(0.81−5.90)	
T3	2.05	(0.77−5.42)	
T1	1.00		0.097
T2 + T3	2.12	(0.87−5.13)	
Continuous^c^	2.74	(0.72−10.42)	0.140
IgM EBV EA-D^d^			
T1	1.00		0.138
T2	1.04	(0.38−2.83)	0.078^e^
T3	2.25	(0.88−5.74)	
T1 + T2	1.00		**0.047**
T3	**2.21**	(1.01−4.82)	
Continuous^c^	3.07	(0.60−15.81)	0.181
IgM N 229E^d^			
T1	1.00		0.147
T2	0.64	(0.24−1.74)	
T3	1.64	(0.67−4.00)	
T1 + T2	1.00		0.074
T3	2.02	(0.93−4.37)	
IgM N HKU1			
T1	1.00		0.128
T2	1.04	(0.40−2.73)	
T3	2.24	(0.91−5.52)	
T1 + T2	1.00		**0.043**
T3	**2.20**	(1.03−4.80)	
Continuous^c^	2.65	(0.66−10.73)	0.172

*This table shows results for the 11 immunoglobulins most related to SARS-CoV-2 seropositivity; results for the other 13 immunoglobulins are shown in Supplementary Table 1. The odds ratios quantify the magnitude of the associations between the exposures and SARS-CoV-2 seropositivity in the 41 SARS-CoV-2 seropositives and the 104 seronegatives. T1 to T3: tertiles. For categorical values of immunoglobulins, ORs shown in bold are ORs ≥ 2.5 or ORs ≤ 0.4 with *p* values <0.05. For continuous values of immunoglobulins, ORs are shown in bold if their *p* values <0.05.

a Unless otherwise specified, odds ratios were adjusted for household outdoor index.

b Unless otherwise specified, *p*-value derived from Wald’s test.

c Odds ratio for each increase of 10 times in the level of the isotype-antigen pair. We present just some examples of statistically nonsignificant continuous variables; all other continuous variables not shown in the table were statistically nonsignificant.

d Odds ratios adjusted for household outdoor index and smoking.

e Multivariate analog of Mantel’s extension test for linear trend.

**Table 4:** Influence of mixtures of isotype-antigen pairs for cytomegalovirus, Epstein–Barr virus, and common cold infections on the risk of SARS-CoV-2 seropositivity (*N* = 145)*.

Model		OR^a^	(95% CI)	*p* ^b^	Model	OR^a^	(95% CI)	*p* ^b^
1a	IgA CMV pp150				1b			
	T1 + T2	1.00		0.004		1.00		0.010
	T3	0.21	(0.07−0.60)			0.27	(0.10−0.73)	
	IgA EBV EA-D							
	T1 + T2	1.00		0.019		1.00		0.016
	T3	3.26	(1.21−8.76)			3.27	(1.24−8.58)	
	IgG N OC43							
	T1 + T2	1.00		0.017		1.00		0.014
	T3	0.30	(0.11−0.80)			0.30	(0.11−0.78)	
	IgM EBV EA-D							
	T1 + T2	1.00		0.021		–		
	T3	2.75	(1.16−6.52)					
2a	IgA CMV pp150				2b			
	T1 + T2	1.00		0.024		1.00		0.025
	T3	0.35	(0.14−0.87)			0.36	(0.15−0.88)	
	IgG EBV VCAp18							
	T1 + T2	1.00		0.070		–		
	T3	0.44	(0.18−1.07)					
	IgG N OC43							
	T1 + T2	1.00		0.044		1.00		0.044
	T3	0.37	(0.14−0.97)			0.38	(0.15−0.98)	
	IgM EBV EA-D							
	T1 + T2	1.00		0.012		1.00		0.018
	T3	3.00	(1.27−7.06)			2.78	(1.19−6.47)	
3a	IgA CMV pp150				3b			
	T1 + T2	1.00		0.012		1.00		0.023
	T3	0.27	(0.10−0.75)			0.32	(0.12−0.86)	
	IgA CMV pp65							
	T1 + T2	1.00		0.012		1.00		0.028
	T3	0.18	(0.05−0.69)			0.25	(0.07−0.86)	
	IgA EBV EA-D							
	T1 + T2	1.00		0.001		1.00		0.004
	T3	10.91	(2.72−43.78)			6.36	(1.79−22.61)	
	IgG N OC43							
	T1 + T2	1.00		0.006		–		
	T3	0.24	(0.09−0.66)					
4a	IgA CMV pp150				4b			
	T1 + T2	1.00		0.003		1.00		0.032
	T3	0.20	(0.07−0.59)			0.38	(0.16−0.92)	
	IgA EBV EA-D							
	T1 + T2	1.00		0.012		–		
	T3	3.61	(1.32−9.86)					
	IgG N OC43							
	T1 + T2	1.00		0.011		1.00		0.035
	T3	0.28	(0.10−0.75)			0.37	(0.15−0.93)	
	IgM CMV pp65							
	T1	1.00		0.029		1.00		0.039
	T2 + T3	3.08	(1.12−8.44)			2.77	(1.05−7.34)	
5a^c^	IgA CMV pp150				5b^c^			
	T1 + T2	1.00		0.005		1.00		0.017
	T3	0.22	(0.08−0.64)			0.30	(0.11−0.80)	
	IgA EBV EA-D							
	T1 + T2	1.00		0.010		1.00		0.013
	T3	3.61	(1.35−9.62)			3.37	(1.30−8.77)	
	IgG CMV pp65							
	T1	1.00		0.019		1.00		0.031
	T2 + T3	0.35	(0.14−0.84)			0.39	(0.17−0.92)	
	IgM EBV EA-D							
	T1 + T2	1.00		0.015		–		
	T3	2.83	(1.22−6.55)					
6a	IgM EBV EA-D				6b			
	T1 + T2	1.00		0.031		1.00		0.055
	T3	2.42	(1.08−5.38)			2.18	(0.99−4.81)	
	IgG CMV pp65							
	T1	1.00		0.050		–		
	T2 + T3	0.44	(0.19−1.00)					
	IgG N OC43							
	T1 + T2	–				1.00		0.049
	T3					0.40	(0.16−1.00)	

*The odds ratios (ORs) quantify the magnitude of the associations between the immunoglobulin isotype-antigen pair and SARS-CoV-2 seropositivity in the 145 individuals, 41 SARS-CoV-2 seropositives and 104 seronegatives. An OR of 1.00 denotes the reference category. T1 to T3: tertiles.

a Unless otherwise specified, odds ratios of the isotype-antigen pair of the immunoglobulins were always mutually adjusted for, and further adjusted by household outdoor index and smoking (both confounders *p* < 0.25).

b Wald’s test (two-tailed).

c Odds ratios of the isotype-antigen pair of the immunoglobulins were mutually adjusted for, and further adjusted by household outdoor index (confounder *p* < 0.25).

Table 4 shows five examples of models of 4 isotype-antigen pairs, each pair being significantly associated to an increased or a decreased risk of seropositivity (models 1a, 2a, 3a, 4a and 5a). None of these five models included more than two of the five individual isotype-antigen pairs individually associated to seropositivity (IgG CMV pp150, IgG CMV pp65, IgG N OC43, IgM CMV pp65, and IgM EBV EA-D). When multi-biomarker models considered only these five individual pairs, only models 6a and 6b included more than one of these five individual pairs. Specifically, the two models show that IgM to EBV EA-D increased the risk of seropositivity, whereas model 6a shows, additionally, a protective effect of IgG to CMV pp65 and model 6b shows also a protective effect of IgG to N OC43. Table 4 also shows examples of models of 3 isotype-antigen pairs, each pair being significantly associated to seropositivity (models 1b, 2b, 3b, 4b, and 5b).

None of the total (non-antigen specific) immunoglobulins, individually or in combination with other total immunoglobulins, was associated with SARS-CoV-2 seropositivity (Supplementary Table 3).

Numerous mixtures of two cytokines with two or three immunoglobulins were associated with SARS-CoV-2 seropositivity (Table 5; Figure 2, column 1. D). Examples of immunoglobulins include the five mentioned above (IgG to CMV pp150, CMV pp65, N OC43, IgM to CMV pp65 and EBV EA-D), as well as IgA to CMV pp150 and EBV EA-D, and IgG to EBV VCAp18 (Figure 2, column 1. D). Their ORs had values similar to when they were analyzed individually and when they were analyzed in mixtures of only cytokines or only immunoglobulins; i.e., again, their effects appeared to be independent of each other.

**Table 5:** Influence of mixtures of cytokines and immunoglobulins on the risk of SARS-CoV-2 seropositivity (*N* = 145)*.

Model		OR^a^	(95% CI)	*p* ^b^	Model	OR^a^	(95% CI)	*p* ^b^
1a	IL-8				1b			
	T1 + T2	1.00		0.004		1.00		0.007
	T3	0.22	(0.08−0.61)			0.25	(0.09−0.68)	
	MIP-1α							
	T1 + T2	1.00		0.004		1.00		0.015
	T3	3.72	(1.51−9.14)			2.91	(1.24−6.85)	
	IgA CMV pp150							
	T1 + T2	1.00		0.013		1.00		0.009
	T3	0.30	(0.12−0.78)			0.29	(0.11−0.73)	
	IgG N OC43							
	T1 + T2	1.00		0.012		–		
	T3	0.27	(0.10−0.75)					
	IgM EBV EA-D							
	T1 + T2	–				1.00		0.031
	T3					2.59	(1.09−6.15)	
2a	IL-8				2b			
	T1 + T2	1.00		0.006		1.00		0.006
	T3	0.24	(0.09−0.67)			0.25	(0.09−0.66)	
	MIP-1α							
	T1 + T2	1.00		0.010		–		
	T3	3.13	(1.31−7.44)					
	IL-15							
	Not quantified	–				1.00		0.048
	Quantified					2.45	(1.01−5.95)	
	IgG CMV pp150							
	T1	1.00		0.055		1.00		0.028
	T2 + T3	0.43	(0.18−1.02)			0.38	(0.16−0.90)	
	IgG N OC43							
	T1 + T2	1.00		0.042		1.00		0.069
	T3	0.36	(0.14−0.97)			0.41	(0.16−1.07)	
3a	MIP-1α				3b			
	T1 + T2	1.00		0.004		1.00		0.008
	T3	3.82	(1.53−9.58)			3.14	(1.34−7.36)	
	G-CSF							
	T1	1.00		0.032		–		
	T2 + T3	0.37	(0.15−0.92)					
	TNF-α							
	T1	–				1.00		0.031
	T2 + T3					0.39	(0.17−0.92)	
	IgG EBV VCAp18							
	T1 + T2	1.00		0.043		1.00		0.049
	T3	0.39	(0.16−0.97)			0.41	(0.17−1.00)	
	IgM EBV EA-D							
	T1 + T2	1.00		0.023		1.00		0.033
	T3	2.66	(1.15−6.16)			2.48	(1.08−5.70)	
4	IL-8							
	T1 + T2	1.00		0.003				
	T3	0.22	(0.08−0.59)					
	IL-17							
	Not quantified	1.00		0.014				
	Quantified	3.11	(1.25−7.69)					
	IgA CMV pp150							
	T1 + T2	1.00		0.010				
	T3	0.29	(0.11−0.75)					
	IgG N OC43							
	T1 + T2	1.00		0.012				
	T3	0.27	(0.10−0.75)					
5a	IL-8				5b			
	T1 + T2	–				1.00		0.005
	T3					0.23	(0.08−0.65)	
	MIP-1α							
	T1 + T2	1.00		0.004		1.00		0.005
	T3	4.08	(1.58−10.50)			3.66	(1.47−9.11)	
	G-CSF							
	T1	1.00		0.044		–		
	T2 + T3	0.38	(0.15−0.97)					
	IgA CMV pp150							
	T1 + T2	1.00		0.026		1.00		0.006
	T3	0.35	(0.14−0.88)			0.25	(0.10−0.67)	
	IgG EBV VCAp18							
	T1 + T2	1.00		0.041		–		
	T3	0.39	(0.15−0.96)					
	IgG N OC43							
	T1 + T2	–				1.00		0.014
	T3					0.27	(0.10−0.77)	
	IgM EBV EA-D							
	T1 + T2	1.00		0.008		1.00		0.038
	T3	3.35	(1.38−8.15)			2.55	(1.05−6.20)	
6a	MIP-1α				6b			
	T1 + T2	1.00		0.003		1.00		0.003
	T3	3.98	(1.61−9.85)			3.97	(1.59−9.87)	
	TNF-α							
	T1	1.00		0.023		1.00		0.038
	T2 + T3	0.35	(0.14−0.86)			0.39	(0.16−0.95)	
	IgA CMV pp150							
	T1 + T2	1.00		0.020		1.00		0.019
	T3	0.32	(0.12−0.84)			0.31	(0.12−0.83)	
	IgG N OC43							
	T1 + T2	1.00		0.009		1.00		0.012
	T3	0.26	(0.10−0.72)			0.27	(0.10−0.75)	
	IgM CMV pp65							
	T1	1.00		0.026		–		
	T2 + T3	3.30	(1.16−9.39)					
	IgM EBV EA-D							
	T1 + T2	–				1.00		0.019
	T3					2.88	(1.19−6.99)	

a Odds ratios of the isotype-antigen pair of the immunoglobulins were always mutually adjusted for, and further adjusted by household outdoor index and smoking (both confounders *p* < 0.25).

b Wald’s test (two-tailed).

*The odds ratios (ORs) quantify the magnitude of the associations between the immunoglobulin isotype-antigen pair and SARS-CoV-2 seropositivity in the 145 individuals, 41 SARS-CoV-2 seropositives and 104 seronegatives. An OR of 1.00 denotes the reference category. T1 to T3: tertiles.

By contrast with what we saw with cytokines above, and as we shall see in more detail in section 3.2, none of five immunoglobulins mentioned above more associated with SARS-CoV-2 seropositivity was also clearly associated with COVID-19, and none of the immunoglobulins more associated with COVID-19 was associated with SARS-CoV-2 seropositivity (Figure 2, columns 1 and 2). While some immunoglobulins increased the risk of seropositivity, no immunoglobulin increased the risk of COVID-19.

Some cytokines were associated with seropositivity in women and not at all in men; notably, EGF (OR in women = 3.4), GM-CSF (OR = 4.9), MCP-1 (OR = 5.5), IL-2 (OR = 4.1) and IFN-*α* (OR = 4.4), (all *p* between 0.01 and 0.04). Others were associated with seropositivity in men and not in women; e.g., MIP-1α (OR in men = 4.6), and TNF-α (OR = 0.3; both *p* < 0.02). Finally, IL-6 was associated with an increased risk of seropositivity in women (OR = 3.5), and with a decreased risk in men (OR = 0.2; both *p* < 0.04).

IgM to N 229E was associated with seropositivity in women and not in men (OR in women = 3.9, *p* < 0.03). Two immunoglobulins were associated with seropositivity in men and not in women: IgG to CMV pp150 (OR in men = 0.3), and IgG to CMV pp65 (OR = 0.3; both *p* < 0.05).

### 3.2 Effects of baseline immune markers on risk of COVID-19

Higher concentrations in 2016–17 of IL-8, TNF-*α*, G-CSF, IL-4, and IL-2R decreased the risk of COVID-19 in 2020–21. IL-8 showed the most marked effect (OR = 0.07, 95% CI: 0.01–0.55), while the other markers had an OR<0.4 (Table 6; Figure 1, columns 2. A and 2. B).

**Table 6:** Effect of individual cytokine levels measured in 2016–17 on the risk of COVID-19 in 2020–21 (*N* = 154)*.

Cytokine	OR^a^	(95% CI)	*p* ^b^
Growth factors			
G-CSF			
T1	1.00		0.121
T2	0.33	(0.09−1.16)	**0.075** ^d^
T3	0.34	(0.09−1.25)	
T1	1.00		**0.040**
T2 + T3	**0.33**	(0.12−0.95)	
Continuous^c^	**0.37**	(0.17−0.83)	**0.016**
EGF			
T1	1.00		0.951
T2	0.84	(0.25−2.83)	
T3	0.86	(0.26−2.83)	
Continuous^c^	1.01	(0.44−2.31)	0.980
FGF			
T1	1.00		0.748
T2	0.69	(0.21−2.28)	
T3	0.66	(0.20−2.20)	
Continuous^c^	0.76	(0.34−1.73)	0.515
GM-CSF			
T1	1.00		0.554
T2	0.52	(0.16−1.72)	
T3	0.71	(0.22−2.34)	
T1	1.00		0.317
T2 + T3	0.60	(0.22−1.63)	
Continuous^c^	0.81	(0.48−1.37)	0.430
HGF			
T1	1.00		0.849
T2	1.18	(0.36−3.89)	
T3	0.83	(0.22−3.14)	
Continuous^c^	0.61	(0.14−2.61)	0.504
VEGF			
T1	1.00		0.737
T2	1.62	(0.48−5.49)	
T3	1.32	(0.36−4.84)	
Continuous^c^	0.82	(0.30−2.23)	0.690
Chemokines			
IL-8			
T1	1.00		0.008
T2	2.74	(0.89−8.42)	
T3	0.12	(0.01−1.01)	
T1 + T2	1.00		**0.012**
T3	**0.07**	(0.01−0.55)	
Continuous^c^	**0.13**	(0.02−0.75)	**0.023**
IP-10			
T1	1.00		0.441
T2	0.86	(0.29−2.60)	0.226^d^
T3	0.40	(0.10−1.67)	
T1 + T2	1.00		0.211
T3	0.43	(0.12−1.61)	
Continuous^c^	0.77	(0.17−3.61)	0.742
RANTES			
T1	1.00		0.355
T2	0.36	(0.09−1.53)	
T3	0.63	(0.21−1.88)	
T1	1.00		0.195
T2 + T3	0.52	(0.19−1.40)	
Continuous^c^	0.39	(0.03−4.87)	0.463
EOTAXIN			
T1	1.00		0.743
T2	1.52	(0.46−5.09)	
T3	1.56	(0.42−5.73)	
Continuous^c^	2.14	(0.14−32.78)	0.586
MIP-1α			
T1	1.00		0.182
T2	0.43	(0.11−1.75)	
T3	1.55	(0.49−4.88)	
T1 + T2	1.00		0.128
T3	2.22	(0.80−6.19)	
Continuous^c^	1.18	(0.60−2.31)	0.637
MIP-1β			
T1	1.00		0.966
T2	1.04	(0.30−3.62)	
T3	1.17	(0.34−4.02)	
Continuous^c^	1.18	(0.52−2.69)	0.687
MCP-1			
T1	1.00		0.198
T2	0.36	(0.09−1.48)	
T3	1.34	(0.42−4.25)	
Continuous^c^	1.14	(0.13−10.03)	0.907
MIG			
Not quantified	1.00		0.949
Quantified	1.03	(0.38−2.84)	
TH1			
IL-2			
T1	1.00		0.591
T2	0.56	(0.16−1.92)	
T3	0.97	(0.29−3.20)	
Continuous^c^	1.01	(0.62−1.66)	0.969
IL-12			
T1	1.00		0.363
T2	2.46	(0.60−10.16)	0.190^d^
T3	2.75	(0.64−11.70)	
T1	1.00		0.159
T2 + T3	2.59	(0.69−9.71)	
Continuous^c^	1.30	(0.31−5.51)	0.719
IFN-γ			
Not quantified	1.00		0.955
Quantified	1.04	(0.26−4.21)	
TH2			
IL-4			
Not quantified	1.00		**0.041**
Quantified	**0.20**	(0.04−0.93)	
IL-5			
Q1 + Q2^e^	1.00		0.215
Q3	1.04	(0.31−3.41)	0.097^d^
Q4	0.16	(0.02−1.28)	
≤Q3	1.00		0.080
Q4	0.16	(0.02−1.25)	
Continuous^c^	0.49	(0.12−1.89)	0.298
IL-13			
T1	1.00		0.642
T2	0.80	(0.24−2.67)	0.347^d^
T3	0.56	(0.17−1.87)	
T1	1.00		0.414
T2 + T3	0.66	(0.25−1.78)	
Continuous^c^	0.76	(0.37−1.56)	0.453
Pro-inflammatory			
IL-1β			
T1	1.00		0.814
T2	1.21	(0.38−3.82)	
T3	0.81	(0.22−2.95)	
Continuous^c^	0.69	(0.27−1.77)	0.442
TNF-α			
T1	1.00		**0.050**
T2	**0.34**	(0.10−1.13)	**0.021** ^d^
T3	**0.23**	(0.06−0.85)	
T1	1.00		**0.016**
T2 + T3	**0.28**	(0.10−0.79)	
Continuous^c^	**0.43**	(0.20−0.90)	**0.025**
IL-6			
T1	1.00		0.556
T2	1.09	(0.34−3.48)	
T3	0.54	(0.16−1.95)	
T1 + T2	1.00		0.283
T3	0.54	(0.18−1.66)	
Continuous^c^	0.71	(0.37−1.35)	0.296
IFN-α			
T1	1.00		0.578
T2	0.59	(0.17−2.10)	
T3	1.15	(0.35−3.80)	
Continuous^c^	0.92	(0.45−1.87)	0.815
IL-2R			
T1	1.00		0.130
T2	0.46	(0.15−1.41)	**0.039** ^d^
T3	0.27	(0.07−1.09)	
T1	1.00		**0.051**
T2 + T3	**0.37**	(0.14−1.00)	
Continuous^c^	**0.37**	(0.15−0.95)	**0.039**
IL-17			
Not quantified	1.00		0.868
Quantified	0.91	(0.31−2.69)	
Regulatory			
IL-7			
T1	1.00		0.512
T2	0.48	(0.14−1.67)	
T3	0.77	(0.24−2.48)	
Continuous^c^	0.85	(0.39−1.83)	0.670
Anti-inflammatory			
IL-10			
Not quantified	1.00		0.158
Quantified	0.49	(0.18−1.32)	
IL-15			
Not quantified	1.00		0.522
Quantified	1.41	(0.49−4.03)	
IL-1RA			
T1	1.00		0.625
T2	0.75	(0.23−2.43)	0.332^d^
T3	0.55	(0.16−1.85)	
T1	1.00		0.387
T2 + T3	0.64	(0.24−1.74)	
Continuous^c^	0.49	(0.15−1.58)	0.235

*The odds ratios quantify the magnitude of the associations between the cytokines’ levels (pg/mL) and COVID-19 in the 20 individuals with COVID-19 and the 134 individuals without the disease. T1 to T3: tertiles. Q1 to Q4: quartiles. For categorical values of cytokines, ORs shown in bold are ORs ≥ 2.5 or ORs ≤ 0.4 with *p* values <0.05. For continuous values of cytokines, ORs are shown in bold if their *p* values <0.05. When we analyzed the risk of COVID-19 disease (vs. no disease), the cytokines IP-10, IL-5, and IL-10 had odds ratios between 0.16 and 0.43, not statistically significantly, in their respective dichotomous forms (T3 vs. T1 + T2 for IP-10, Q4 vs. ≤Q3 for IL-5, and quantified vs. not quantified for IL-10). Table 1 and this table are the only parts of the article in which all 30 cytokines appear, thus including cytokines that are not associated with the respective outcomes, SARS-CoV-2 seropositivity and COVID-19 disease.

a Odds ratios were always adjusted for age, smoking, and educational level.

b Unless otherwise specified, *p*-value derived from Wald’s test.

c Odds ratio for each increase of 10 times in the level of the cytokine or factor.

d Multivariate analog of Mantel’s extension test for linear trend.

e The category is exclusively made up of individuals whose cytokine level was less than the respective lower limit of quantification (see Methods, section 2.3).

Most cytokines in mixtures with IL-8, TNF-α, MIP-1α, and G-CSF were associated with COVID-19 (Table 7). Again, in most models MIP-1α had an OR>2.0, and IL-8, TNF-α, and G-CSF had an OR<0.4 (all *p* < 0.05). IL-2R, IL-4, and IL-5 also lowered the risk of the disease. There was no association of IL-6 with COVID-19, nor with SARS-CoV-2 seropositivity, even when IL-6 was considered jointly with IL-8 and TNF-*α*.

**Table 7:** Influence of mixtures of cytokines on the risk of COVID-19 (*N* = 154)*.

Model		OR^a^	(95% CI)	*p* ^b^	Model	OR^a^	(95% CI)	*p* ^b^
1a	IL-8				1b			
	T1 + T2	–				1.00		0.010
	T3					0.05	(0.01−0.50)	
	MIP-1α							
	T1 + T2	1.00		0.016		1.00		0.019
	T3	4.05	(1.30−12.66)			4.41	(1.28−15.25)	
	IL-5							
	≤Q3	1.00		0.048		1.00		0.023
	Q4	0.11	(0.01−0.98)			0.07	(0.01−0.69)	
	TNF-α							
	T1	1.00		0.011		1.00		0.029
	T2 + T3	0.24	(0.08−0.72)			0.27	(0.08−0.87)	
2a	IL-8				2b^c^			
	T1 + T2	1.00		0.020		1.00		0.007
	T3	0.08	(0.01−0.67)			0.05	(0.01−0.44)	
	MIP-1α							
	T1 + T2	1.00		0.007		1.00		0.078
	T3	6.84	(1.70−27.58)			3.15	(0.88−11.32)	
	IL-5							
	≤Q3	–				1.00		0.049
	Q4					0.11	(0.01−0.99)	
	IL-2R							
	T1	1.00		0.092		1.00		0.049
	T2 + T3	0.34	(0.10−1.19)			0.29	(0.09−1.00)	
	IL-4							
	Not quantified	1.00		0.040		–		
	Quantified	0.14	(0.02−0.92)					
	IL-12							
	T1	–				1.00		0.144
	T2 + T3					2.84	(0.70−11.55)	
3a	IL-8				3b^c^			
	T1 + T2	1.00		0.022		1.00		0.018
	T3	0.08	(0.01−0.69)			0.08	(0.01−0.65)	
	MIP-1α							
	T1 + T2	1.00		0.006		1.00		0.028
	T3	7.19	(1.78−29.05)			4.04	(1.16−14.04)	
	G-CSF							
	T1	1.00		0.084		1.00		0.018
	T2 + T3	0.31	(0.08−1.17)			0.21	(0.06−0.79)	
	IL-4							
	Not quantified	1.00		0.046		–		
	Quantified	0.15	(0.02−0.96)					

*The odds ratios (ORs) quantify the magnitude of the associations between the exposures and COVID-19 in the 154 individuals, 20 with COVID-19 and 134 without the disease. An OR of 1.00 denotes the reference category. T1 to T3: tertiles. Q1 to Q4: quartiles.

a Odds ratios of the cytokines were mutually adjusted for, and further adjusted by age, education, and smoking (all three confounders *p* < 0.25).

b Wald’s test (two-tailed).

c Odds ratios of the cytokines were mutually adjusted for, and further adjusted by education (confounder *p* < 0.25).

The four cytokines most consistently associated with the risk of COVID-19 (G-CSF, IL-8, TNF-α, and MIP-1α) were also associated with the risk of seropositivity and associations were in the same direction (Figure 1, column 2. C).

Generally, complementary models (Figure 1, columns 2. E to 2. G; Supplementary Table 1.2) were coherent with logistic regression models for IL-8, TNF-α, G-CSF, and IL-2R (Figure 1, columns 2. A to 2. D).

The four isotype-antigen pairs more strongly associated with risk of COVID-19 (all protective) were IgA to CMV pp65 and N 229E, IgG to EBV EA-D, and IgG to EBV VCAp18 (Table 8; Supplementary Table 4; Figure 2, columns 2. A and 2. B).

**Table 8:** Effect of selected individual isotype-antigen pairs for cytomegalovirus, Epstein–Barr virus, and common cold infections measured in 2016–17 on the risk of COVID-19 in 2020–21 (*N* = 154)*.

Isotype-antigen pair	OR^a^	(95% CI)	*p* ^b^
IgA CMV pp150			
T1	1.00		0.109
T2	1.62	(0.49−5.42)	
T3	0.37	(0.08−1.77)	
T1 + T2	1.00		0.053
T3	0.27	(0.07−1.02)	
Continuous^c^	0.21	(0.03−1.70)	0.144
IgA CMV pp65			
T1	1.00		0.111
T2	0.31	(0.09−1.07)	
T3	0.35	(0.10−1.22)	
T1	1.00		**0.036**
T2 + T3	**0.33**	(0.12−0.93)	
Continuous^c^	0.41	(0.06−2.63)	0.348
IgA EBV EA-D			
T1	1.00		0.157
T2	0.27	(0.07−1.07)	
T3	0.56	(0.17−1.79)	
T1	1.00		0.075
T2 + T3	0.40	(0.15−1.10)	
Continuous^c^	0.12	(0.01−1.18)	0.069
IgA N 229E			
T1	1.00		0.099
T2	0.35	(0.10−1.18)	0.052^d^
T3	0.30	(0.09−1.05)	
T1	1.00		**0.032**
T2 + T3	**0.33**	(0.12−0.91)	
Continuous^c^	**0.27**	(0.08−0.99)	**0.048**
IgG EBV EA-D^e^			
T1	1.00		0.065
T2	**0.30**	(0.09−1.01)	**0.044** ^d^
T3	**0.30**	(0.09−0.99)	
T1	1.00		**0.019**
T2 + T3	**0.30**	(0.11−0.82)	
Continuous^c^	0.15	(0.01−1.58)	0.113
IgG EBV VCAp18			
T1	1.00		0.123
T2	0.77	(0.25−2.35)	**0.042** ^d^
T3	**0.24**	(0.06−0.95)	
T1 + T2	1.00		**0.047**
T3	**0.26**	(0.07−0.98)	
Continuous^c^	**0.23**	(0.06−0.94)	**0.041**

*This table shows results for the 6 immunoglobulins most related to COVID-19; results for the other 18 immunoglobulins are shown in Supplementary Table 4. The odds ratios quantify the magnitude of the associations between the exposures and COVID-19 in the 20 individuals with COVID-19 and the 134 individuals without the disease. T1 to T3: tertiles. For categorical values of immunoglobulins, ORs shown in bold are ORs ≥ 2.5 or ORs ≤ 0.4 with *p* values <0.05. For continuous values of immunoglobulins, ORs are shown in bold if their *p* values <0.05.

a Unless otherwise specified, odds ratios were adjusted for age, smoking, and educational level.

b Unless otherwise specified, *p*-value derived from Wald’s test.

c Odds ratio for each increase of 10 times in the level of the isotype-antigen pair.

d Multivariate analog of Mantel’s extension test for linear trend.

e Odds ratios adjusted for age and smoking.

These four isotype-antigen pairs, as well as IgA to EBV EA-D and N OC43, and IgG to CMV pp150 were part of mixtures associated with COVID-19 (all protective), with most ORs between 0.2 and 0.4 (all *p* ≤ 0.03; Table 9; Figure 2, column 2. C).

**Table 9:** Influence of mixtures of isotype-antigen pairs for cytomegalovirus, Epstein–Barr virus, and common cold infections on the risk of COVID-19 (*N* = 154)*.

Model		OR^a^	(95% CI)	*p* ^b^	Model	OR^a^	(95% CI)	*p* ^b^
1a^c^	IgA CMV pp150				1b^c^			
	T1 + T2	1.00		0.159		–		
	T3	0.35	(0.08−1.51)					
	IgA CMV pp65							
	T1	1.00		0.020		1.00		0.008
	T2 + T3	0.26	(0.08−0.81)			0.22	(0.07−0.67)	
	IgG CMV pp150							
	T1 + T2	1.00		0.027		1.00		0.016
	T3	0.20	(0.05−0.84)			0.18	(0.04−0.73)	
	IgG EBV VCAp18							
	T1 + T2	1.00		0.023		1.00		0.022
	T3	0.19	(0.05−0.80)			0.19	(0.05−0.79)	
2a^c^	IgA CMV pp65				2b^c^			
	T1	1.00		0.027		–		
	T2 + T3	0.27	(0.09−0.86)					
	IgA N OC43							
	T1	1.00		0.142		1.00		0.035
	T2 + T3	0.42	(0.13−1.34)			0.31	(0.10−0.92)	
	IgG CMV pp150							
	T1 + T2	1.00		0.013		1.00		0.021
	T3	0.16	(0.04−0.68)			0.19	(0.05−0.78)	
	IgG EBV VCAp18							
	T1 + T2	1.00		0.018		1.00		0.024
	T3	0.18	(0.04−0.75)			0.21	(0.05−0.81)	
3a	IgA N 229E				3b^c^			
	T1	1.00		0.038		–		
	T2 + T3	0.31	(0.10−0.94)					
	IgG CMV pp150							
	T1 + T2	1.00		0.024		1.00		0.016
	T3	0.19	(0.05−0.80)			0.18	(0.04−0.73)	
	IgG EBV VCAp18							
	T1 + T2	1.00		0.044		1.00		0.022
	T3	0.24	(0.06−0.96)			0.19	(0.05−0.79)	
	IgA CMV pp65							
	T1	–				1.00		0.008
	T2 + T3					0.22	(0.07−0.67)	
4a	IgA N OC43				4b			
	T1	1.00		0.040		–		
	T2 + T3	0.31	(0.10−0.95)					
	IgG CMV pp150							
	T1 + T2	1.00		0.022		1.00		0.029
	T3	0.20	(0.05−0.79)			0.21	(0.05−0.85)	
	IgG EBV VCAp18							
	T1 + T2	1.00		0.019		1.00		0.020
	T3	0.19	(0.05−0.76)			0.19	(0.05−0.77)	
	IgA EBV EA-D							
	T1	–				1.00		0.049
	T2 + T3					0.34	(0.12−1.00)	

*The odds ratios (ORs) quantify the magnitude of the associations between the immunoglobulin isotype-antigen pair and COVID-19 in the 154 individuals, 20 with COVID-19 and 134 without the disease. An OR of 1.00 denotes the reference category. T1 to T3: tertiles.

a Unless otherwise specified, Odds ratios of the isotype-antigen pair of the immunoglobulins were mutually adjusted for, and further adjusted by age, education and smoking (all confounders *p* < 0.25).

b Wald’s test (two-tailed).

c Odds ratios of the isotype-antigen pair of the immunoglobulins were mutually adjusted for, and further adjusted by education and smoking (both confounders *p* < 0.25).

Among total immunoglobulins, only IgG1, IgG3, and IgA were marginally associated with COVID-19, with ORs ≤ 0.4 (Supplementary Table 5). Because of low statistical power, there were no mixtures of two or more total immunoglobulins significantly associated with COVID-19, in spìte of ORs near 0.4 (Supplementary Table 6).

Remarkably, mixtures of cytokines and immunoglobulins associated with COVID-19 included between two to four cytokines and one to two immunoglobulins. Examples include: IL-8, MIP-1*α*, TNF-α, and IL-2R with IgA to CMV pp150 and N 229E, and IgG to EBV EA-D (Table 10; Figures 1, 2, sections 2. D). Cytokines and immunoglobulins associated with COVID-19 were always associated in the same direction (lowering or increasing risk) whether they were individually analyzed, analyzed in exclusive mixtures of cytokines or immunoglobulins, or as mixtures of cytokines and immunoglobulins. Furthermore, some cytokines as MIP-1α had ORs increased up to 3 times when they were included in mixtures compared to when they were considered as single biomarkers (see, for instance, Tables 6, 7, 10).

**Table 10:** Influence of mixtures of cytokines and immunoglobulins on the risk of COVID-19 (*N* = 154)*.

Model		OR^a^	(95% CI)	*p* ^b^	Model	OR^a^	(95% CI)	*p* ^b^
1a	IL-8				1b			
	T1 + T2	1.00		0.009		1.00		0.015
	T3	0.06	(0.01−0.49)			0.07	(0.01−0.59)	
	MIP1-α							
	T1 + T2	1.00		0.035		1.00		0.033
	T3	3.67	(1.09−12.34)			4.01	(1.12−14.35)	
	TNF-α							
	T1	1.00		0.033		–		
	T2 + T3	0.28	(0.09−0.90)					
	IL-2R							
	T1	–				1.00		0.047
	T2 + T3					0.28	(0.08−0.99)	
	IgA CMV pp150							
	T1 + T2	1.00		0.016		–		
	T3	0.16	(0.04−0.71)					
	IgA N 229E							
	T1	–				1.00		0.043
	T2 + T3					0.29	(0.09−0.96)	
2a	MIP-1α				2b			
	T1 + T2	1.00		0.009		1.00		0.017
	T3	5.78	(1.56−21.45)			5.07	(1.34−19.21)	
	IL-4							
	Not quantified	1.00		0.004		–		
	Quantified	0.07	(0.01−0.45)					
	IL-2R							
	T1	–				1.00		0.008
	T2 + T3					0.17	(0.05−0.64)	
	IgA N 229E							
	T1	1.00		0.026		1.00		0.030
	T2 + T3	0.26	(0.08−0.85)			0.28	(0.09−0.88)	
	IgG CMV pp150							
	T1 + T2	1.00		0.017		1.00		0.027
	T3	0.15	(0.03−0.71)			0.19	(0.04−0.83)	
3a	IL-8				3b			
	T1 + T2	1.00		0.010		1.00		0.014
	T3	0.06	(0.01−0.50)			0.06	(0.01−0.58)	
	MIP-1α							
	T1 + T2	1.00		0.015		1.00		0.028
	T3	5.15	(1.37−19.36)			4.27	(1.17−15.55)	
	G-CSF							
	T1	1.00		0.026		1.00		0.046
	T2 + T3	0.22	(0.06−0.83)			0.26	(0.07−0.98)	
	IgA CMV pp150							
	T1 + T2	1.00		0.017		–		
	T3	0.16	(0.04−0.72)					
	IgA N 229E							
	T1	–				1.00		0.040
	T2 + T3					0.29	(0.09−0.95)	
4a	IL-8				4b			
	T1 + T2	1.00		0.015		1.00		0.015
	T3	0.07	(0.01−0.60)			0.07	(0.01−0.59)	
	MIP-1α							
	T1 + T2	1.00		0.023		1.00		0.032
	T3	4.50	(1.23−16.46)			3.68	(1.12−12.12)	
	TNF-α							
	T1	–				1.00		0.016
	T2 + T3					0.23	(0.07−0.76)	
	IL-2R							
	T1	1.00		0.009		–		
	T2 + T3	0.18	(0.05−0.66)					
	IgG CMV pp150							
	T1 + T2	1.00		0.032		–		
	T3	0.19	(0.04−0.87)					
	IgG EBV EA-D							
	T1	–				1.00		0.015
	T2 + T3					0.21	(0.06−0.74)	
5a	IL-8				5b			
	T1 + T2	1.00		0.013		–		
	T3	0.05	(0.01−0.54)					
	MIP-1α							
	T1 + T2	1.00		0.030		1.00		0.005
	T3	3.94	(1.14−13.57)			6.83	(1.78−26.27)	
	TNF-α							
	T1	1.00		0.048		1.00		0.029
	T2 + T3	0.29	(0.08−0.99)			0.27	(0.08−0.87)	
	IL-4							
	Not quantified	–				1.00		0.008
	Quantified					0.08	(0.01−0.52)	
	IgA N 229E							
	T1	1.00		0.037		1.00		0.046
	T2 + T3	0.26	(0.07−0.92)			0.29	(0.08−0.98)	
	IgG EBV EA-D							
	T1	1.00		0.018		–		
	T2 + T3	0.21	(0.06−0.77)					
	IgG CMV pp150							
	T1 + T2	–				1.00		0.025
	T3					0.16	(0.03−0.80)	

Model		OR^a^	(95% CI)	*p* ^b^
6	IL-8			
	T1 + T2	1.00		0.006
	T3	0.04	(0.00−0.39)	
	MIP-1α			
	T1 + T2	1.00		0.009
	T3	5.69	(1.53−21.17)	
	IL-5			
	≤Q3	1.00		0.019
	Q4	0.06	(0.01−0.64)	
	TNF-α			
	T1	1.00		0.020
	T2 + T3	0.22	(0.06−0.79)	
	IgG EBV EA-D			
	T1	1.00		0.013
	T2 + T3	0.18	(0.05−0.70)	
7	RANTES			
	T1	1.00		0.031
	T2 + T3	0.27	(0.08−0.89)	
	IL-12			
	T1	1.00		0.029
	T2 + T3	5.06	(1.18−21.69)	
	IL-5			
	≤Q3	1.00		0.016
	Q4	0.07	(0.01−0.60)	
	IgG CMV pp150			
	T1 + T2	1.00		0.014
	T3	0.16	(0.04−0.70)	
	IgG EBV VCAp18			
	T1	1.00		0.032
	T2 + T3	0.28	(0.09−0.90)	

*The odds ratios (ORs) quantify the magnitude of the associations between the immunoglobulin isotype-antigen pair and COVID-19 in the 154 individuals, 20 with COVID-19 and 134 without the disease. An OR of 1.00 denotes the reference category. T1 to T3: tertiles.

a Odds ratios were always mutually adjusted for, and further adjusted by age, education and smoking (all confounders p < 0.25).

b Wald’s test (two-tailed).

Two cytokines were associated with COVID-19 in women and not in men: G-CSF (OR in women = 0.1), and IL-2R (OR = 0.2; both *p* < 0.05). And two were associated with COVID-19 in men and not in women: MIP-1α (OR in men = 7.0), and TNF-α (OR = 0.2; both *p* ≤ 0.02); this was similarly observed above for seropositivity (section 3.1).

Two immunoglobulins were associated with COVID-19 in women and not in men; IgA to CMV pp65 (OR = 0.2) and IgM to N OC43 (OR = 0.1; both *p* < 0.04). IgA to N 229E was associated with COVID-19 among men and not in women (OR in men = 0.1, *p* < 0.01).

The associations of cytokines and immunoglobulins with seropositivity and COVID-19 were not consistently stronger in older than in younger age groups.

When we considered comorbidities previously found slightly associated with some immunoglobulins (8), we found that they did not change the results just shown above. For instance, when dyslipidemia was included in models assessing the associations between levels of the biomarkers of inflammation and of immunological status, and the two outcomes (SARS-CoV-2 seropositivity and COVID-19), the estimates did not change.

## 4 Discussion

### 4.1 Assessment of main findings

Well into the pandemic, in late 2021—and still today, to a large extent—the capacity of pre-existing immunity to HCoV to crossprotect against *de novo* COVID-19 was largely unknown. So was also the possible influence of the basal immune state, analyzed here through cytokines and immunoglobulins, on the risk of SARS-CoV-2 infection and COVID-19. The unique longitudinal design of the present study, with measurements before and during the pandemic, provides novel knowledge on the protective and deleterious effects of specific individual cytokines and immunoglobulins, and their mixtures.

We previously reported intraindividual stability between prepandemic (2016–17) and pandemic (2020–21) levels of cytokines and immunoglobulins, including antibodies against HCoV. Furthermore, the stability was similar in study participants who in 2020–21 were SARS-CoV-2 seropositive and seronegative, and between participants who did and did not develop COVID-19 (8). The intraindividual stability suggests that SARS-CoV-2 infection may not boost anti-HCoV N responses, although cross-reactivity has been suggested in other studies (22–24). These results are in line with findings suggesting no cross-reactive neutralizing activity against SARS-CoV-2 in 37 prepandemic sera samples from Edinburgh hospital patients with prior seasonal coronavirus infection (25, 26). In the sera of 76 healthy French donors, no anti-RBD reactivity was detected, although six samples were found to be reactive against one or several of the other SARS-CoV-2 antigens: except for these six samples, pre-existing immunity to HCoV was not responsible for recall-type IgG responses to SARS-CoV-2, and it did not lead to cross-protection against COVID-19 (27). A general upward trend in anti-HCoV N antibody levels was observed in 33 health care workers from a hospital in Barcelona when comparing levels prior to and after SARS-CoV-2 infection. For instance, IgG to 229E significantly increased after SARS-CoV-2 seroconversion. However, not all seroconverters had an increase in levels, supporting a back-boost of N HCoV beyond cross-reactivity (15). In the present study, IgA to OC43 and 229E and IgG to OC43 were associated to a lower risk of COVID-19; the latter was also associated with lower risk of SARS-CoV-2 infection (seropositivity). IgA and IgG to CMV and EBV were associated with lower risk of COVID-19. IgA to CMV and IgG to CMV and EBV were also associated with a lower risk of infection. Previous studies have observed cross-reactive antibody responses against SARS-CoV-2 spike protein in prepandemic samples (28, 29) and some could be protective. In fact, crossreactivity of endemic common cold human coronaviruses and CMV with SARS-CoV-2 has been associated to lower risk of COVID-19 (30–32).

In agreement with our observation of the association of IgG to N OC43 with lower risk of SARS-CoV-2 seropositivity, a recent study with transgenic mice shows that human coronavirus OC43-elicited CD4^+^ T cells may protect against SARS-CoV-2 (33). Also in agreement with our observation of the association of IgG to CMV with lower risk of SARS-CoV-2 seropositivity and lower risk of COVID-19, previous studies show that CMV seropositivity and T cell responses associate with SARS-CoV-2 cellular and serological responses (34, 35) suggesting crossreactivity that contributes to the pre-existing immunity against SARS-CoV-2 (29). The association of some antibodies with a reduced risk of disease and others with a reduced risk of infection, suggests different levels of cross-reactivity, some controlling viral load after the infection and some blocking viral entry into host cells.

In our study we report an association of high levels of IgM to EBV EA-D and CMV pp65 in prepandemic samples (which are suggestive of viral reactivation) with a higher risk of SARS-CoV-2 infection. EBV and CMV can reactivate in immunocompromised individuals, as well as in the setting of physiologic stressors. Thus, reactivation of these viruses in prepandemic samples is indicative of higher vulnerability in these subjects in front of new infections. This would explain the association observed in the present study between IgM to EBV EA-D and CMV pp65 in prepandemia samples and SARS-coV-2 seropositivity.

The finding that higher IgG1 and IgG3 basal levels may lower risk of COVID-19 is consistent with their higher effector capacity against pathogens compared to IgG2 and IgG4 (36). The association of higher IgA basal serum levels (which correlate with mucosal levels) with a lower risk of COVID-19 is consistent with its important role protecting from infections that target mucosal tissues. A previous study has also shown that total serum IgA levels are negatively associated with the severity of COVID-19 (37).

Some cytokines had consistent and clear associations with SARS-CoV-2 infection and COVID-19, among them TNF-*α* and IL-8, protective in both instances. TNF-α is produced by macrophages and monocytes and is one of the early effectors that alert the host’s immunity about dangers. When SARS-CoV-2 reaches the bronchial epithelia, TNF-α is induced, promoting the infiltration of macrophages, dendritic cells, natural killer cells, and neutrophils to the bronchi to control and clear SARS-CoV-2 replication (38). IL-8, also induced in the bronchial epithelia by SARS-CoV-2 (39), is a potent chemotactic factor that attracts neutrophils, basophils, and T-cells during the inflammatory process. High basal blood levels of these two cytokines may induce a more efficient local innate immune response in the respiratory system that blocks SARS-CoV-2 replication, preventing the virus to reach the lymph nodes so there is no seropositivization or disease.

We observed that high MIP-1α basal levels increased the risk of SARS-CoV-2 infection and COVID-19. MIP-1α is a chemokine involved mainly in cell adhesion and migration. Severe COVID-19 has been associated with significantly higher MIP-1α (40). This chemokine is implicated in the autocrine regulation of migration of dendritic cells to draining lymph nodes (41–43). The association of high levels of MIP-α with a higher risk of seropositivity and COVID-19 could be related to a higher migration of virus-infected dendritic cells facilitating virus spread, skew of T-cell responses through altered cytokine production, and induction of apoptosis in T cells leading to immunosuppression (44).

Higher prepandemic concentrations of IL-2R, IL-4 and IL-5 protected against COVID-19, but not against SARS-CoV-2 infection. These findings suggest a role for these cytokines in the regulation of the inflammatory response under infection. Besides the reported role of IL-5 in helping antibody production by B cells in mice, there are also evidences of this role in humans; for example, Huston et al. (45) showed that human B cells express IL-5 receptor mRNA and respond to IL-5 with enhanced IgM production after mitogenic stimulation. IL-4 and IL-5 are involved in promoting a Th2 immune response, which helps in antibody production by B cells. IL-4 acts as a potent B cell growth factor—enhancing proliferation, survival, and class-switch recombination toward IgG1 and IgE—while IL-5 reinforces Th2-mediated antibody responses and is co-secreted by an IL-5^+^ subset of Th2 cells (46). Thus, in the context of COVID-19, IL-4 and IL-5 may contribute to enhancing humoral immunity, promoting the generation of antibodies that target the SARS-CoV-2 virus. IL-5 also influences eosinophils that can play a protective role by helping clear viral infection. Balanced IL-4 and IL-5 responses could aid prevent exaggerated inflammation, thus reducing the risk of COVID-19.

### 4.2 Study limitations and strengths

The availability of two biological measurements for each individual participant, one before and one after the pandemic onset, is a major strength of the study. Therefore, the time sequence is clear: cytokines and immunoglobulins were measured in blood samples collected 4 years before the two outcomes (SARS-CoV-2 seropositivity and COVID-19). While this feature is unique in the literature on the pandemic, it is essential to assess causes, mediators, and effects. Our study design avoids biases common with prevalent cases of undefined origin and cross-sectional studies. The population-based design is also a strength: it is less prone to bias than studies that recruit patients attending an Emergency Department or a primary care center, or admitted to hospital. Nevertheless, confirmation of our findings in larger populations with different characteristics than ours and exposed to different SARS-CoV-2 types is required.

We previously showed that intraindividual changes in cytokines and immunoglobulins between 2016–17 and 2020–21 were moderate (8). As mentioned above, we showed that the stability was similar between participants who in 2020–21 were SARS-CoV-2 seropositive and seronegative, and between participants who did and did not develop COVID-19 (8). The similarity has methodological relevance for the present paper: it indicates that it is valid to use prepandemic levels of cytokines and immunoglobulins to assess their risk relationship (protective or harmful) with the development of SARS-CoV-2 seropositivity and COVID-19.

It is difficult to attribute a viral infection, which depends on various risk factors such as exposure, behavior, and comorbidities, to an inflammatory profile measured 4 years before the viral exposure. The association could be influenced by unassessed variables and other conditions that might interfere with the immune response. The multifactorial nature of SARS-CoV-2 infection, with factors such as viral load, comorbidities, and environmental factors, needs to be considered. Our results show an association between specific cytokines measured before the pandemic and the risk of seropositivity (infection) and COVID-19 (disease). This suggests that the immunological status before exposure affects susceptibility to infection and disease. The relationship between cytokines and risk of infection may not be direct, cytokines could be a surrogate marker of the immune status. The observed effect of the cytokines (immune status) would be independent of the behavior and exposure to the virus. While the immune status and cytokine profile seem to be stable over time [as observed in our study (8) and others], they may be influenced by environmental factors and comorbidities, which may also directly increase susceptibility of infection independently of the cytokine profile.

In our study participants levels of cytokines and immunoglobulins in late 2019 (i.e., the time closest to the pandemic outbreak) were putatively well correlated with their levels in 2016–2017 (8). Nevertheless, new studies could improve on such periods (encompassing the last months of 2019, expanding the study during the pandemic and even after its conclusion) with currently stored but yet unused data. This aim seems feasible, for instance, with existing population-based cohort studies that include biobanks. We remain hopeful that such biological samples and clinico-epidemiological data will undergo the much needed analyses (6, 7).

We could assess selection biases [as previously defined (47)] and, if they existed, seem unlikely to explain the associations observed. If something, the associations might be underestimated, because the 66 subjects who did not attend the follow-up visit were likely more susceptible to the outcomes than the 174 participants (6). As common in clinical and population research in the real world, our criteria to define COVID-19 disease (section 2.6.2.) do not have 100% sensitivity and specificity. Yet, we think the analysis of the two outcomes provides valid and relevant estimates of the associations with the levels of cytokines and immunoglobulins.

The selection of the immunological parameters analyzed was guided by their biological relevance, our prior experience, and their alignment with the study objectives. We selected a panel of 30 cytokines, chemokines and growth factors that represented the mediators produced by the main immune cell families, including Th1, Th2, Th17, and both pro-inflammatory, anti-inflammatory/regulatory functions. This breadth of pathways is well suited to evaluate baseline immune status comprehensively. Moreover, this particular 30-plex assay has been evaluated for its performance in relation to other leading commercial kits by the ISGlobal group, subsequently optimized, and widely used in multiple studies previous to this one, for assessing infection, vaccination and baseline cytokine levels in many types of cohorts and patients.

In addition to the cytokines, the Antibody Isotyping 7-Plex Human ProcartaPlex™ panel was chosen because it enables a detailed assessment of the overall humoral immunity, and it has also been employed in previous studies to investigate baseline and disease-related immunoglobulin profiles.

The inclusion of human cold coronavirus antigens in the serology Luminex panel was based on the reported cross-reactivity with the nucleocapside from SARS-CoV-2 to assess association of previous exposure with risk of SARS-CoV-2 infection. The inclusion of antigens from herpes virus in the Luminex panel was based on their reported immunomodulatory effects.

Therefore, all the measurements included had an underlying scientific reason. With state-of-the-art techniques (13–16), we analyzed 30 cytokines, 24 isotype-antigen pairs, and 7 total immunoglobulins, a relatively large amount in itself, common in the clinical literature, yet not usually easy to measure in a real human cohort from the general population; this was even more difficult in the pandemic times of 2020–21. We could thus perform a considerable number of comparisons. Since ours is the first study assessing the influence of cytokines and immunoglobulins on the risks of the two outcomes in a general, non-institutionalized population, it is only reasonable that we assessed comprehensively such associations. Certainly, these features of the study may generate false positives (and replication or refutation of our findings in larger studies is required); but they have also strengths, since the number of candidates (i.e., potentially relevant cytokines and immunoglobulins) is high. The models could barely be based on clinical and epidemiological evidence on cytokines, immunoglobulins and SARS-CoV-2 and COVID-19 in a non-institutionalized population, because little evidence of this sort is available.

Also, we detected more associations than expected by chance, and many went in the direction of decreasing risks, whereas more positive associations (increased risks) would be expected by chance. There is no consensus on techniques to adjust for the number of comparisons in clinical and epidemiological studies, and such techniques may have low efficiency or poor accuracy (18). Thus, the statistical tests and confidence intervals were not adjusted for multiple testing, and should not be used to infer definitive effects. We consider the priority given to detect potential associations as warranted as long as the results inspire larger population-based, prospective studies and laboratory research. Indeed, as sketched in 4.1., the results should encourage translational research from the observations we made in a real human population to the clinic and the laboratory; that is, they can inspire further clinical and laboratory research on mechanisms through which cytokines and immunoglobulins may influence immune processes and contribute to SARS-CoV-2 seropositivity and COVID-19. Perhaps, as mediators of some of the environmental contaminants that we tentatively identified (6).

Since the study population was modest, the statistical power and precision were often low. Yet, numerous effect estimates were statistically significant, mostly when the OR was ≥2 or OR≤0.4. Because of statistical power, there were no mixtures of two or more total immunoglobulins significantly associated with COVID-19; such mixtures would likely be statistically significant in larger studies. Also due to low numbers—only two of 20 COVID-19 cases had been hospitalized and the rest were of moderate severity—, we could not assess the association of cytokines and immunoglobulins with the severity of the infection and the severity of disease, on vaccine response, and on persistent COVID-19. Our ongoing follow-up and subject accrual may overcome these weaknesses.

While in Tables 1, 3, 6, 8, we provide a number of results of tertile analysis of cytokines and immunoglobulins, we often also dichotomized such exposures, given the common absence of a linear dose–response or lack of evidence on influential levels. Sometimes, the lack of linear dose-responses in tertile analyses coexisted with substantial odds ratios in some tertiles, thus indicating again that the conduct of independent analyses in larger populations is necessary.

While some interactions between pairs of cytokines and immunoglobulins could be biologically plausible and relevant, we were again cautioned by the small size of our current study population, and do not present results. Neither do we for other interactions with personal and social characteristics (except sex), which also deserve to be tested in larger human studies. Cytokine profiles in males and females exhibit notable differences due to hormonal influences, which may underlie the sex-specific cytokines (IL-6, MIP-1α, G-CSF) associated with SARS-CoV-2 infection or COVID-19 (48, 49). Our analyses considered the whole population of 154 persons who were at risk for infection, rather than only the seropositives at risk for COVID-19, for clear methodological reasons, previously explained (6).

## 5 Conclusion

The unique longitudinal design of this study, with measurements before and during the pandemic in a general population, provides novel knowledge on the protective and detrimental effects of specific individual cytokines and immunoglobulins, and their mixtures, on the risk of SARS-CoV-2 seropositivity and COVID-19. The results deserve to be refuted or replicated in existing population-based cohort studies with biobanks. If confirmed, findings would be significantly relevant for medicine and public health.

## Data Availability

The raw data supporting the conclusions of this article may be made available by the authors upon reasonable request, without undue reservation.

## References

[1] Menges D Zens KD Ballouz T Caduff N Llanas-Cornejo D Aschmann HE. Heterogenous humoral and cellular immune responses with distinct trajectories post-SARS-CoV-2 infection in a population-based cohort. Nat Commun. (2022) 13:4855. doi: 10.1038/s41467-022-32573-w, PMID: 35982045 PMC9386650

[2] Le Bert N Chia WN Wan WY AKJ T Chong SZ Tan N. Widely heterogeneous humoral and cellular immunity after mild SARS-CoV-2 infection in a homogeneous population of healthy young men. Emerg Microbes Infect. (2021) 10:2141–50. doi: 10.1080/22221751.2021.1999777, PMID: 34709140 PMC8604544

[3] Mazzoni A Maggi L Capone M Vanni A Spinicci M Salvati L. Heterogeneous magnitude of immunological memory to SARS-CoV-2 in recovered individuals. Clin Transl Immunol. (2021) 10:e1281. doi: 10.1002/cti2.1281, PMID: 33976879 PMC8101693

[4] Souquette A Thomas PG. Variation in the basal immune state and implications for disease. eLife. (2024) 13:e90091. doi: 10.7554/eLife.90091, PMID: 38275224 PMC10817719

[5] Karachaliou M Moncunill G Espinosa A Castaño-Vinyals G Jiménez A Vidal M. Infection induced SARS-CoV-2 seroprevalence and heterogeneity of antibody responses in a general population cohort study in Catalonia Spain. Sci Rep. (2021) 11:21571. doi: 10.1038/s41598-021-00807-4, PMID: 34732749 PMC8566562

[6] Porta M Pumarega J Gasull M Aguilar R Henríquez-Hernández LA Basagaña X. Individual blood concentrations of persistent organic pollutants and chemical elements, and COVID-19: a prospective cohort study in Barcelona. Environ Res. (2023) 223:115419. doi: 10.1016/j.envres.2023.115419 36740154 PMC9898057

[7] Pumarega J Gasull M Koponen J Campi L Rantakokko P Henríquez-Hernández LA. Prepandemic personal concentrations of per- and polyfluoroalkyl substances (PFAS) and other pollutants: specific and combined effects on the incidence of COVID-19 disease and SARS-CoV-2 infection. Environ Res. (2023) 237:116965. doi: 10.1016/j.envres.2023.116965, PMID: 37652221

[8] Gasull M Pumarega J Aguilar R Campi L Prieto-Merino D Villar-García J. Stability of cytokine and immunoglobulin concentrations in the general population: prepandemic basal concentrations and intraindividual changes until the COVID-19 pandemic. Front Public Health. (2025) 13: 1548379. doi: 10.3389/fpubh.2025.1548379 40672932 PMC12263939

[9] Del Valle DM Kim-Schulze S Huang HH Beckmann ND Nirenberg S Wang B. An inflammatory cytokine signature predicts COVID-19 severity and survival. Nat Med. (2020) 26:1636–43. doi: 10.1038/s41591-020-1051-9, PMID: 32839624 PMC7869028

[10] Cervia C Zurbuchen Y Taeschler P Ballouz T Menges D Hasler S. Immunoglobulin signature predicts risk of post-acute COVID-19 syndrome. Nat Commun. (2022) 13:446. doi: 10.1038/s41467-021-27797-1, PMID: 35078982 PMC8789854

[11] Porta M Pumarega J Henríquez-Hernández LA Gasull M Bartoll X Arrebola JP. Reductions in blood concentrations of persistent organic pollutants in the general population of Barcelona from 2006 to 2016. Sci Total Environ. (2021) 777:146013. doi: 10.1016/j.scitotenv.2021.146013

[12] Gasull M Camargo J Pumarega J Henríquez-Hernández LA Campi L Zumbado M. Blood concentrations of metals, essential trace elements, rare earth elements and other chemicals in the general adult population of Barcelona: distribution and associated sociodemographic factors. Sci Total Environ. (2024) 909:168502. doi: 10.1016/j.scitotenv.2023.168502, PMID: 37977377

[13] Pons MJ Gomes C Aguilar R Barrios D Aguilar-Luis MA Ruiz J. Immunosuppressive and angiogenic cytokine profile associated with *Bartonella bacilliformis* infection in post-outbreak and endemic areas of carrion’s disease in Peru. PLoS Negl Trop Dis. (2017) 11:e0005684. doi: 10.1371/journal.pntd.0005684, PMID: 28628613 PMC5491314

[14] Rubio R Aguilar R Bustamante M Muñoz E Vázquez-Santiago M Santano R. Maternal and neonatal immune response to SARS-CoV-2, IgG transplacental transfer and cytokine profile. Front Immunol. (2022) 13:999136. doi: 10.3389/fimmu.2022.999136, PMID: 36238312 PMC9552073

[15] Ortega N Ribes M Vidal M Rubio R Aguilar R Williams S. Seven-month kinetics of SARS-CoV-2 antibodies and role of pre-existing antibodies to human coronaviruses. Nat Commun. (2021) 12:4740. doi: 10.1038/s41467-021-24979-9, PMID: 34362897 PMC8346582

[16] Dobaño C Vidal M Santano R. Highly sensitive and specific multiplex antibody assays to quantify immunoglobulins M, a, and G against SARS-CoV-2 antigens. J Clin Microbiol. (2020) 59:e01731. doi: 10.1128/JCM.01731-20 PMC8111153 33127841

[17] World Health Organization (WHO). Public health surveillance for COVID-19 interim guidance. (2022). WHO/2019-nCoV/SurveillanceGuidance/2022.1. Available online at: https://www.who.int/publications/i/item/WHO-2019-nCoV-SurveillanceGuidance-2022.1 (Accessed June 2, 2025).

[18] Lash TL VanderWeele TJ Haneuse S Rothman KJ, eds. Modern epidemiology. 4th. ed., Philadelphia: Wolters-Kluwer, (2021): 390–392.

[19] Porta M. Greenland S. Hernán M. eds. A dictionary of epidemiology. 6th. edition. New York: Oxford University Press and International Epidemiological Association, (2014): 261–262.

[20] Julian P Helsel D (2021). NADA2: data analysis for censored environmental data. R package version 1.0.2.

[21] Dunn PK Smyth GK. Generalized linear models with examples in R. New York: Springer (2018).

[22] Dobaño C Santano R Jiménez A. Immunogenicity and crossreactivity of antibodies to the nucleocapsid protein of SARS-CoV-2: utility and limitations in seroprevalence and immunity studies. Transl Res. (2021) 232:60–74. doi: 10.1016/j.trsl.2021.02.006, PMID: 33582244 PMC7879156

[23] Abela IA Schwarzmüller M Ulyte A Radtke T Haile SR Ammann P. Cross-protective HCoV immunity reduces symptom development during SARS-CoV-2 infection. MBio. (2024) 15:e0272223. doi: 10.1128/mbio.02722-23, PMID: 38270455 PMC10865973

[24] Murray SM Ansari AM Frater J Klenerman P Dunachie S Barnes E. The impact of pre-existing cross-reactive immunity on SARS-CoV-2 infection and vaccine responses. Nat Rev Immunol. (2023) 23:304–16. doi: 10.1038/s41577-022-00809-x, PMID: 36539527 PMC9765363

[25] Poston D Weisblum Y Wise H Templeton K Jenks S Hatziioannou T. Absence of severe acute respiratory syndrome coronavirus 2 neutralizing activity in Prepandemic sera from individuals with recent seasonal coronavirus infection. Clin Infect Dis. (2021) 73:e1208–11. doi: 10.1093/cid/ciaa1803, PMID: 33270134 PMC7799301

[26] Lee M. Lack of severe acute respiratory syndrome coronavirus 2 neutralization by antibodies to seasonal coronaviruses: making sense of the coronavirus disease 2019 pandemic. Clin Infect Dis. (2021) 73:e1212–3. doi: 10.1093/cid/ciab011, PMID: 34492701 PMC7929053

[27] Miyara M Saichi M Sterlin D Anna F Marot S Mathian A. Pre-COVID-19 immunity to common cold human coronaviruses induces a recall-type IgG response to SARS-CoV-2 antigens without cross-neutralisation. Front Immunol. (2022) 13:790334. doi: 10.3389/fimmu.2022.790334, PMID: 35222375 PMC8873934

[28] Jaago M Rähni A Pupina N Pihlak A Sadam H Tuvikene J. Differential patterns of cross-reactive antibody response against SARS-CoV-2 spike protein detected for chronically ill and healthy COVID-19 naïve individuals. Sci Rep. (2022) 12:16817. doi: 10.1038/s41598-022-20849-6, PMID: 36207326 PMC9540097

[29] Pothast CR Dijkland RC Thaler M Hagedoorn RS Kester MGD Wouters AK. SARS-CoV-2-specific CD4+ and CD8+ T cell responses can originate from cross-reactive CMV-specific T cells. eLife. (2022) 11:e82050. doi: 10.7554/eLife.82050, PMID: 36408799 PMC9822249

[30] Lipsitch M Grad YH Sette A Crotty S. Cross-reactive memory T cells and herd immunity to SARS-CoV-2. Nat Rev Immunol. (2020) 20:709–13. doi: 10.1038/s41577-020-00460-4, PMID: 33024281 PMC7537578

[31] Aran D Beachler DC Lanes S Overhage JM. Prior presumed coronavirus infection reduces COVID-19 risk: a cohort study. J Inf Secur. (2020) 81:923–30. doi: 10.1016/j.jinf.2020.10.023, PMID: 33127456 PMC7590640

[32] Sagar M Reifler K Rossi M Miller NS Sinha P White LF. Recent endemic coronavirus infection is associated with less-severe COVID-19. J Clin Invest. (2021) 131:e143380. doi: 10.1172/JCI143380, PMID: 32997649 PMC7773342

[33] Dos Santos Alves RP Timis J Miller R. Human coronavirus OC43-elicited CD4+ T cells protect against SARS-CoV-2 in HLA transgenic mice. Nat Commun. (2024) 15:787. doi: 10.1038/s41467-024-45043-2 38278784 PMC10817949

[34] Frozza FTB Fazolo T de Souza PO Lima K da Fontoura JC Borba TS. A high CMV-specific T cell response associates with SARS-CoV-2-specific IL-17 T cell production. Med Microbiol Immunol. (2023) 212:75–91. doi: 10.1007/s00430-022-00758-1, PMID: 36512097 PMC9745694

[35] Jo N Zhang R Ueno H Yamamoto T Weiskopf D Nagao M. Aging and CMV infection affect pre-existing SARS-CoV-2-reactive CD8+ T cells in unexposed individuals. Front Aging. (2021) 2:719342. doi: 10.3389/fragi.2021.719342, PMID: 35822004 PMC9261342

[36] Damelang T Brinkhaus M van Osch TLJ Schuurman J Labrijn AF Rispens T. Impact of structural modifications of IgG antibodies on effector functions. Front Immunol. (2024) 14:1304365. doi: 10.3389/fimmu.2023.1304365, PMID: 38259472 PMC10800522

[37] Barzegar-Amini M Mahmoudi M Dadgarmoghaddam M Farzad F Najafabadi AQ Jabbari-Azad F. Comparison of serum total IgA levels in severe and mild COVID-19 patients and control group. J Clin Immunol. (2022) 42:10–8. doi: 10.1007/s10875-021-01149-6, PMID: 34694544 PMC8542492

[38] Mohd Zawawi Z Kalyanasundram J Mohd Zain R Thayan R Basri DF Yap WB. Prospective roles of tumor necrosis factor-alpha (TNF-α) in COVID-19: prognosis, therapeutic and management. Int J Mol Sci. (2023) 24:6142. doi: 10.3390/ijms24076142, PMID: 37047115 PMC10094668

[39] Gasparello J d’Aversa E Breveglieri G Borgatti M Finotti A Gambari R. In vitro induction of interleukin-8 by SARS-CoV-2 spike protein is inhibited in bronchial epithelial IB3-1 cells by a miR-93-5p agomiR. Int Immunopharmacol. (2021) 101:108201. doi: 10.1016/j.intimp.2021.108201, PMID: 34653729 PMC8492649

[40] Hamza AM Ali WDK Hassanein N Albassam WB Barry M AlFaifi AMM. Relation between macrophage inflammatory protein-1 and intercellular adhesion molecule-1 and computed tomography findings in critically-ill saudi covid-19 patients. J Infect Public Health. (2022) 15:1497–502. doi: 10.1016/j.jiph.2022.10.023, PMID: 36423464 PMC9617641

[41] Dieu-Nosjean MC Vicari A Lebecque S Caux C. Regulation of dendritic cell trafficking: a process that involves the participation of selective chemokines. J Leukoc Biol. (1999) 66:252–62. doi: 10.1002/jlb.66.2.252, PMID: 10449163

[42] Sozzani S Allavena P Vecchi A Mantovani A. Chemokines and dendritic cell traffic. J Clin Immunol. (2000) 20:151–60. doi: 10.1023/A:1006659211340, PMID: 10941822

[43] Sallusto F Lanzavecchia A. Understanding dendritic cell and T-lymphocyte traffic through the analysis of chemokine receptor expression. Immunol Rev. (2000) 177:134–40. doi: 10.1034/j.1600-065X.2000.17717.x, PMID: 11138771

[44] Larsson M Beignon AS Bhardwaj N. DC-virus interplay: a double edged sword. Semin Immunol. (2004) 16:147–61. doi: 10.1016/j.smim.2004.02.002, PMID: 15130499

[45] Huston MM Moore JP Mettes HJ Tavana G Huston DP. Human B cells express IL-5 receptor messenger ribonucleic acid and respond to IL-5 with enhanced IgM production after mitogenic stimulation with *Moraxella catarrhalis*. J Immunol. (1996) 156:1392–401. doi: 10.4049/jimmunol.156.4.1392, PMID: 8568239

[46] Upadhyaya B Yin Y Hill BJ Douek DC Prussin C. Hierarchical IL-5 expression defines a subpopulation of highly differentiated human Th2 cells. J Immunol. (2011) 187:3111–20. doi: 10.4049/jimmunol.1101283, PMID: 21849680 PMC3445433

[47] Porta M Gasull M Puigdomènech E Rodríguez-Sanz M Pumarega J Rebato C. Sociodemographic factors influencing participation in the Barcelona health survey study on serum concentrations of persistent organic pollutants. Chemosphere. (2009) 76:216–25. doi: 10.1016/j.chemosphere.2009.03.030, PMID: 19386342

[48] Klein SL Flanagan KL. Sex differences in immune responses. Nat Rev Immunol. (2016) 16:626–38. doi: 10.1038/nri.2016.90, PMID: 27546235

[49] Takahashi T Ellingson MK Wong P Israelow B Lucas C Klein J. Sex differences in immune responses that underlie COVID-19 disease outcomes. Nature. (2020) 588:315–20. doi: 10.1038/s41586-020-2700-3, PMID: 32846427 PMC7725931

