# Supplementary material for: Prepandemic levels of cytokines and immunoglobulins and risk of SARS-CoV-2 infection and COVID-19 in the general population of Barcelona
Source: Front Public Health. 2025 Aug 20;13:1548456. doi: 10.3389/fpubh.2025.1548456 (PMC12405241; doi:10.3389/fpubh.2025.1548456)
Supplement: Supplementary file 1 [file Data_Sheet_1.docx]

**Supplementary material**

**Supplementary Table 1.1.** Complementary regression models* of the relationship

between cytokine levels and SARS-CoV-2 seropositivity (N = 145)

| **Cytokine** | **Ratio**^a^ | **(95% CI)** | | **P**^b^ |  |
| --- | --- | --- | --- | --- | --- |
| **G-CSF** |  |  |  |  |  |
| Linear regression model | 0.58 | (0.31 | -1.10) | 0.100 |  |
|  |  |  |  |  |  |
| **IL-8** |  |  |  |  |  |
| Linear regression model | 0.86 | (0.78 | -0.95) | 0.003 |  |
| Quasi-binomial regression | 0.75 | (0.60 | -0.93) | 0.010 |  |
|  |  |  |  |  |  |
| **MIP-1α** |  |  |  |  |  |
| Regression for censored data | 1.68 | (0.86 | -3.27) | 0.126 |  |
| Quasi-binomial regression | 1.38 | (0.99 | -1.91) | 0.058 |  |
|  |  |  |  |  |  |
| **TNF-α** |  |  |  |  |  |
| Linear regression model | 0.60 | (0.34 | -1.06) | 0.081 |  |
| Regression for censored data | 0.57 | (0.31 | -1.03) | 0.064 |  |
|  |  |  |  |  |  |
| **IL-17** |  |  |  |  |  |
| Linear regression model | 1.62 | (1.08 | -2.43) | 0.021 |  |
| Regression for censored data | 2.21 | (0.88 | -5.54) | 0.092 |  |
| Quasi-binomial regression | 1.71 | (0.88 | -3.34) | 0.117 |  |
|  |  |  |  |  | |

^a^ Ratios of the association between each cytokine and seropositivity adjusted for outdoor household index.

^b^ Wald’s test (two-tailed).

*Examples of results of the complementary regression models: 1) linear regression with each cytokine (in the log 10 scale) as the continuous response and the seropositivity (or COVID-19) as the main explanatory variable, with the corresponding adjustment covariables; 2) regression methods for censored data using the NADA2 library in R; and 3) normalised cytokine values as a proportion between 0 and 1 of the limits of quantification (value - lLOQ) / (uLOQ - lLOQ), and comparison of this ratio between groups with a quasi-binomial generalised linear regression. See Methods (section 2.7). The main results of the three models are shown in columns E to G of Figure 1 and column E of Figure 2.

Supplementary material of:

Porta M, Pumarega J, Aguilar R, Prieto-Merino D, Campi L, Rius C, Villar-García J, Vidal M, Jimenez A, Peña A, Muñoz MA, Trasande L, Bolúmar F, Moncunill G, Gasull M, Dobaño C. Prepandemic levels of cytokines and immunoglobulins and risk of SARS-CoV-2 infection and COVID-19 in the general population of Barcelona. *Frontiers in Public Health* 2025; 13: 1548456. <https://doi.org/10.3389/fpubh.2025.1548456>. Preprint posted on 24 Oct 2024 <https://www.researchsquare.com/article/rs-5117251/v1>.

**Supplementary Table 1.2.** Complementary regression models of relationship

between cytokines and COVID-19 (N = 154)

| **Cytokine** | **Ratio**^a^ | **(95% CI)** | | **P**^b^ |
| --- | --- | --- | --- | --- |
| **G-CSF** |  |  |  |  |
| Linear regression model | 0.37 | (0.16 | -0.84) | 0.019 |
| Regression for censored data | 0.35 | (0.14 | -0.90) | 0.030 |
|  |  |  |  |  |
| **IL-8** |  |  |  |  |
| Linear regression model | 0.70 | (0.52 | -0.93) | 0.014 |
| Quasi-binomial regression | 0.84 | (0.73 | -0.96) | 0.010 |
|  |  |  |  |  |
| **IL-4** |  |  |  |  |
| Regression for censored data | 0.39 | (0.14 | -1.11) | 0.077 |
|  |  |  |  |  |
| **TNF-α** |  |  |  |  |
| Linear regression model | 0.41 | (0.19 | -0.89) | 0.024 |
| Regression for censored data | 0.42 | (0.20 | -0.87) | 0.021 |
| Quasi-binomial regression | 0.61 | (0.38 | -0.97) | 0.038 |
|  |  |  |  |  |
| **IL-2R** |  |  |  |  |
| Linear regression model | 0.47 | (0.23 | -0.95) | 0.037 |
| Quasi-binomial regression | 0.64 | (0.45 | -0.91) | 0.013 |
|  |  |  |  |  |

^a^ Ratios of the association between each cytokine and COVID-19 adjusted for age, education and smoking.

^b^ Wald’s test (two-tailed).

See also notes to Supplementary Table 1.1.

**Supplementary Table 2.** Effect of other individual isotype-antigen pairs for cytomegalovirus, Epstein-Barr virus, and common cold infections measured in 2016-17 on the risk of SARS-CoV-2 seropositivity in 2020-21 (N=145)*

| **Isotype-antigen pair** | **OR^a^** | **(95% CI)** | | **P^b^** |  |
| --- | --- | --- | --- | --- | --- |
|  |  |  |  |  |  |
| **IgA EBV VCAp18** |  |  |  |  |  |
| T1 | 1.00 |  |  | 0.635 |  |
| T2 | 0.95 | (0.37 | -2.39) |  |  |
| T3 | 1.41 | (0.57 | -3.49) |  |  |
|  |  |  |  |  |  |
| T1+T2 | 1.00 |  |  | 0.344 |  |
| T3 | 1.45 | (0.67 | -3.13) |  |  |
|  |  |  |  |  |  |
| **IgA N 229E**^d^ |  |  |  |  |  |
| T1 | 1.00 |  |  | 0.384 |  |
| T2 | 0.51 | (0.20 | -1.34) |  |  |
| T3 | 0.81 | (0.33 | -1.98) |  |  |
|  |  |  |  |  |  |
| T1 | 1.00 |  |  | 0.297 |  |
| T2+T3 | 0.65 | (0.29 | -1.45) |  |  |
|  |  |  |  |  |  |
| **IgA N HKU1**^d^ |  |  |  |  |  |
| T1 | 1.00 |  |  | 0.443 |  |
| T2 | 1.68 | (0.67 | -4.21) |  |  |
| T3 | 1.01 | (0.38 | -2.65) |  |  |
|  |  |  |  |  |  |
| continuous^c^ | 1.93 | (0.58 | -6.45) | 0.286 |  |
|  |  |  |  |  |  |
| **IgA N NL63** |  |  |  |  |  |
| T1 | 1.00 |  |  | 0.424 |  |
| T2 | 0.56 | (0.23 | -1.39) |  |  |
| T3 | 0.66 | (0.27 | -1.60) |  |  |
|  |  |  |  |  |  |
| **IgA N OC43** |  |  |  |  |  |
| T1 | 1.00 |  |  | 0.512 |  |
| T2 | 0.89 | (0.35 | -2.29) |  |  |
| T3 | 1.47 | (0.60 | -3.60) |  |  |
|  |  |  |  |  |  |
| **IgG EBV EA-D**^d^ |  |  |  |  |  |
| T1 | 1.00 |  |  | 0.462 |  |
| T2 | 0.56 | (0.22 | -1.42) |  |  |
| T3 | 0.70 | (0.28 | -1.74) |  |  |
|  |  |  |  |  |  |
| T1 | 1.00 |  |  | 0.248 |  |
| T2+T3 | 0.63 | (0.22 | -1.42) |  |  |
|  |  |  |  |  |  |
| **IgG N 229E**^d^ |  |  |  |  |  |
| T1 | 1.00 |  |  | 0.220 |  |
| T2 | 0.44 | (0.16 | -1.18) |  |  |
| T3 | 0.90 | (0.37 | -2.17) |  |  |
|  |  |  |  |  |  |
| **IgG N HKU1** |  |  |  |  |  |
| T1 | 1.00 |  |  | 0.371 |  |
| T2 | 0.60 | (0.24 | -1.46) | 0.202 | ^e^ |
| T3 | 0.55 | (0.22 | -1.37) |  |  |
|  |  |  |  |  |  |
| T1 | 1.00 |  |  | 0.161 |  |
| T2+T3 | 0.57 | (0.26 | -1.25) |  |  |
|  |  |  |  |  |  |
| **IgG N NL63** |  |  |  |  |  |
| T1 | 1.00 |  |  | 0.780 |  |
| T2 | 0.89 | (0.36 | -2.23) |  |  |
| T3 | 0.73 | (0.30 | -1.78) |  |  |
|  |  |  |  |  |  |

[ continued ]

**Supplementary Table 2**, continued.

| **Isotype-antigen pair** | **OR^a^** | **(95% CI)** | | **P^b^** |  |
| --- | --- | --- | --- | --- | --- |
|  |  |  |  |  |  |
| **IgM CMV pp150** |  |  |  |  |  |
| T1 | 1.00 |  |  | 0.582 |  |
| T2 | 0.69 | (0.26 | -1.78) |  |  |
| T3 | 1.09 | (0.45 | -2.63) |  |  |
|  |  |  |  |  |  |
| **IgM EBV VCAp18** |  |  |  |  |  |
| T1 | 1.00 |  |  | 0.960 |  |
| T2 | 1.04 | (0.43 | -2.56) |  |  |
| T3 | 1.14 | (0.46 | -2.82) |  |  |
|  |  |  |  |  |  |
| **IgM N NL63** |  |  |  |  |  |
| T1 | 1.00 |  |  | 0.972 |  |
| T2 | 0.89 | (0.35 | -2.28) |  |  |
| T3 | 0.95 | (0.39 | -2.35) |  |  |
|  |  |  |  |  |  |
| **IgM N OC43**^d^ |  |  |  |  |  |
| T1 | 1.00 |  |  | 0.255 |  |
| T2 | 1.96 | (0.77 | -5.00) |  |  |
| T3 | 1.04 | (0.39 | -2.78) |  |  |
|  |  |  |  |  |  |

* This Table shows results for the 13 immunoglobulins least related to SARS-CoV-2 seropositivity; results for the other 11 immunoglobulins are shown in Table 3. The odds ratios quantify the magnitude of the associations between the exposures and SARS-CoV-2 seropositivity in the 41 SARS-CoV-2 seropositives and the 104 seronegatives.

T1 to T3: tertiles.

^a^ Unless otherwise specified, odds ratios were adjusted for household outdoor index.

^b^ Unless otherwise specified, *p*‑value derived from Wald’s test.

^c^ Odds ratio for each increase of 10 times in the level of the isotype-antigen pair. We present just some examples of statistically nonsignificant continuous variables; all other continuous variables not shown in the Table were statistically nonsignificant.

^d^ Odds ratios adjusted for household outdoor index and smoking.

^e^ Multivariate analogue of Mantel’s extension test for linear trend.

**Supplementary Table 3.** Effect of total immunoglobulins measured in 2016-17

on the risk of SARS-CoV-2 seropositivity in 2020-21 (N=145)*

| **Immunoglobulin** | **OR^a^** | **(95% CI)** | | **P^b^** |  |
| --- | --- | --- | --- | --- | --- |
|  |  |  |  |  |  |
| **Ig G1** |  |  |  |  |  |
| T1 | 1.00 |  |  | 0.610 |  |
| T2 | 0.67 | (0.25 | -1.75) |  |  |
| T3 | 1.02 | (0.43 | -2.45) |  |  |
|  |  |  |  |  |  |
| continuous^c^ | 1.14 | (0.14 | -9.39) | 0.904 |  |
|  |  |  |  |  |  |
| **Ig G2**^d^ |  |  |  |  |  |
| T1 | 1.00 |  |  | 0.591 |  |
| T2 | 0.63 | (0.24 | -1.69) |  |  |
| T3 | 0.98 | (0.40 | -2.37) |  |  |
|  |  |  |  |  |  |
| T1 | 1.00 |  |  | 0.615 |  |
| T2+T3 | 0.81 | (0.37 | -1.81) |  |  |
|  |  |  |  |  |  |
| continuous^c^ | 0.34 | (0.02 | -5.87) | 0.459 |  |
|  |  |  |  |  |  |
| **Ig G3** |  |  |  |  |  |
| T1 | 1.00 |  |  | 0.862 |  |
| T2 | 0.79 | (0.31 | -2.02) |  |  |
| T3 | 0.81 | (0.33 | -1.97) |  |  |
|  |  |  |  |  |  |
| continuous^c^ | 0.57 | (0.05 | -6.50) | 0.648 |  |
|  |  |  |  |  |  |
| **Ig G4** |  |  |  |  |  |
| T1 | 1.00 |  |  | 0.189 |  |
| T2 | 2.03 | (0.77 | -5.32) | 0.083 | ^e^ |
| T3 | 2.36 | (0.91 | -6.11) |  |  |
|  |  |  |  |  |  |
| T1 | 1.00 |  |  | 0.073 |  |
| T2+T3 | 2.19 | (0.93 | -5.15) |  |  |
|  |  |  |  |  |  |
| continuous^c^ | 2.23 | (0.80 | -6.22) | 0.125 |  |
|  |  |  |  |  |  |
| **Sum IgGs** |  |  |  |  |  |
| T1 | 1.00 |  |  | 0.937 |  |
| T2 | 0.91 | (0.35 | -2.41) |  |  |
| T3 | 1.08 | (0.45 | -2.61) |  |  |
|  |  |  |  |  |  |
| continuous^c^ | 0.78 | (0.05 | -12.81) | 0.863 |  |
|  |  |  |  |  |  |
| **Ig E** |  |  |  |  |  |
| T1 | 1.00 |  |  | 0.378 |  |
| T2 | 1.77 | (0.68 | -4.60) | 0.210 | ^e^ |
| T3 | 1.83 | (0.73 | -4.57) |  |  |
|  |  |  |  |  |  |
| T1 | 1.00 |  |  | 0.164 |  |
| T2+T3 | 1.80 | (0.79 | -4.12) |  |  |
|  |  |  |  |  |  |
| continuous^c^ | 2.34 | (0.60 | -9.18) | 0.223 |  |
|  |  |  |  |  |  |
| **Ig A** |  |  |  |  |  |
| T1 | 1.00 |  |  | 0.988 |  |
| T2 | 0.98 | (0.40 | -2.37) | 0.878 | ^e^ |
| T3 | 0.93 | (0.36 | -2.40) |  |  |
|  |  |  |  |  |  |
| continuous^c^ | 0.88 | (0.09 | -8.53) | 0.912 |  |
|  |  |  |  |  |  |

[ continued ]

**Supplementary Table 3**, continued.

| **Immunoglobulin** | **OR^a^** | **(95% CI)** | | **P^b^** |  |
| --- | --- | --- | --- | --- | --- |
|  |  |  |  |  |  |
| **Ig M**^d^ |  |  |  |  |  |
| T1 | 1.00 |  |  | 0.477 |  |
| T2 | 0.77 | (0.28 | -2.09) |  |  |
| T3 | 1.37 | (0.56 | -3.40) |  |  |
|  |  |  |  |  |  |
| continuous^c^ | 3.05 | (0.48 | -19.31) | 0.236 |  |
|  |  |  |  |  |  |

* The odds ratios quantify the magnitude of the associations between the immunoglobin isotypes and subclasses concentrations (µg/mL), and SARS-CoV-2 seropositivity in the 41 SARS-CoV-2 seropositives and the 104 seronegatives.

T1 to T3: tertiles.

^a^ Unless otherwise specified, odds ratios were adjusted for household outdoor index. ^b^ Unless otherwise specified, *p*‑value derived from Wald’s test. ^c^ Odds ratio for each increase of 10 times in the level of the immunoglobin isotypes and subclasses.

^d^ Odds ratios were further adjusted for smoking.

^e^ Multivariate analogue of Mantel’s extension test for linear trend.

**Supplementary Table 4.** Effect of other individual isotype-antigen pairs for cytomegalovirus, Epstein-Barr virus, and common cold infections measured in 2016-17 on the risk of COVID-19 in 2020-21 (N=154)*

| **Isotype-antigen pair** | **OR^a^** | **(95% CI)** | | **P^b^** |  |
| --- | --- | --- | --- | --- | --- |
|  |  |  |  |  |  |
| **IgA EBV VCAp18** |  |  |  |  |  |
| T1 | 1.00 |  |  | 0.289 |  |
| T2 | 0.45 | (0.14 | -1.48) | 0.163 | ^d^ |
| T3 | 0.41 | (0.12 | -1.47) |  |  |
|  |  |  |  |  |  |
| T1 | 1.00 |  |  | 0.116 |  |
| T2+T3 | 0.43 | (0.15 | -1.23) |  |  |
|  |  |  |  |  |  |
| continuous^c^ | 0.38 | (0.06 | -2.53) | 0.317 |  |
|  |  |  |  |  |  |
| **IgA N HKU1** |  |  |  |  |  |
| T1 | 1.00 |  |  | 0.653 |  |
| T2 | 0.64 | (0.19 | -2.15) | 0.399 | ^d^ |
| T3 | 0.61 | (0.18 | -2.03) |  |  |
|  |  |  |  |  |  |
| **IgA N NL63** |  |  |  |  |  |
| T1 | 1.00 |  |  | 0.648 |  |
| T2 | 0.60 | (0.18 | -2.04) |  |  |
| T3 | 0.65 | (0.20 | -2.07) |  |  |
|  |  |  |  |  |  |
| continuous^c^ | 0.57 | (0.21 | -1.56) | 0.271 |  |
|  |  |  |  |  |  |
| **IgA N OC43** |  |  |  |  |  |
| T1 | 1.00 |  |  | 0.277 |  |
| T2 | 0.45 | (0.14 | -1.48) | 0.150 | ^d^ |
| T3 | 0.40 | (0.11 | -1.45) |  |  |
|  |  |  |  |  |  |
| continuous^c^ | 0.47 | (0.10 | -2.19) | 0.339 |  |
|  |  |  |  |  |  |
| **IgG CMV pp150** |  |  |  |  |  |
| T1 | 1.00 |  |  | 0.147 |  |
| T2 | 0.73 | (0.24 | -2.26) | 0.052 | ^d^ |
| T3 | 0.24 | (0.06 | -1.01) |  |  |
|  |  |  |  |  |  |
| T1+T2 | 1.00 |  |  | 0.061 |  |
| T3 | 0.28 | (0.07 | -1.06) |  |  |
|  |  |  |  |  |  |
| continuous^c^ | 0.48 | (0.17 | -1.33) | 0.158 |  |
|  |  |  |  |  |  |
| **IgG CMV pp65** |  |  |  |  |  |
| T1 | 1.00 |  |  | 0.450 |  |
| T2 | 0.45 | (0.13 | -1.59) |  |  |
| T3 | 0.79 | (0.24 | -2.62) |  |  |
|  |  |  |  |  |  |
| **IgG N 229E** |  |  |  |  |  |
| T1 | 1.00 |  |  | 0.682 |  |
| T2 | 0.79 | (0.21 | -2.96) |  |  |
| T3 | 1.35 | (0.42 | -4.33) |  |  |
|  |  |  |  |  |  |
| **IgG N HKU1** |  |  |  |  |  |
| T1 | 1.00 |  |  | 0.646 |  |
| T2 | 0.82 | (0.26 | -2.65) | 0.355 | ^d^ |
| T3 | 0.55 | (0.16 | -1.93) |  |  |
|  |  |  |  |  |  |
| **IgG N NL63** |  |  |  |  |  |
| T1 | 1.00 |  |  | 0.552 |  |
| T2 | 0.50 | (0.15 | -1.75) |  |  |
| T3 | 0.69 | (0.22 | -2.18) |  |  |
|  |  |  |  |  |  |

[ continued ]

**Supplementary Table 4**, continued.

| **Isotype-antigen pair** | **OR^a^** | **(95% CI)** | | **P^b^** |  |
| --- | --- | --- | --- | --- | --- |
|  |  |  |  |  |  |
| **IgG N OC43** |  |  |  |  |  |
| T1 | 1.00 |  |  | 0.265 |  |
| T2 | 1.20 | (0.41 | -3.47) |  |  |
| T3 | 0.32 | (0.06 | -1.64) |  |  |
|  |  |  |  |  |  |
| T1 | 1.00 |  |  | 0.112 |  |
| T2+T3 | 0.29 | (0.06 | -1.34) |  |  |
|  |  |  |  |  |  |
| continuous^c^ | 0.25 | (0.05 | -1.21) | 0.085 |  |
|  |  |  |  |  |  |
| **IgM CMV pp150** |  |  |  |  |  |
| T1 | 1.00 |  |  | 0.336 |  |
| T2 | 0.37 | (0.09 | -1.42) |  |  |
| T3 | 0.58 | (0.18 | -1.92) |  |  |
|  |  |  |  |  |  |
| **IgM CMV pp65** |  |  |  |  |  |
| T1 | 1.00 |  |  | 0.327 |  |
| T2 | 1.82 | (0.49 | -6.81) |  |  |
| T3 | 0.74 | (0.18 | -2.96) |  |  |
|  |  |  |  |  |  |
| continuous^c^ | 0.42 | (0.06 | -2.93) | 0.383 |  |
|  |  |  |  |  |  |
| **IgM EBV EA-D** |  |  |  |  |  |
| T1 | 1.00 |  |  | 0.469 |  |
| T2 | 0.46 | (0.11 | -1.81) |  |  |
| T3 | 0.97 | (0.30 | -3.12) |  |  |
|  |  |  |  |  |  |
| **IgM EBV VCAp18** |  |  |  |  |  |
| T1 | 1.00 |  |  | 0.671 |  |
| T2 | 1.11 | (0.30 | -4.07) |  |  |
| T3 | 0.65 | (0.16 | -2.59) |  |  |
|  |  |  |  |  |  |
| **IgM N 229E** |  |  |  |  |  |
| T1 | 1.00 |  |  | 0.166 |  |
| T2 | 0.23 | (0.05 | -1.06) |  |  |
| T3 | 0.52 | (0.16 | -1.73) |  |  |
|  |  |  |  |  |  |
| continuous^c^ | 0.42 | (0.08 | -2.32) | 0.321 |  |
|  |  |  |  |  |  |
| **IgM N HKU1** |  |  |  |  |  |
| T1 | 1.00 |  |  | 0.763 |  |
| T2 | 0.62 | (0.17 | -2.29) |  |  |
| T3 | 0.74 | (0.22 | -2.49) |  |  |
|  |  |  |  |  |  |
| continuous^c^ | 0.37 | (0.05 | -2.64) | 0.320 |  |
|  |  |  |  |  |  |
| **IgM N NL63** |  |  |  |  |  |
| T1 | 1.00 |  |  | 0.472 |  |
| T2 | 0.47 | (0.13 | -1.75) |  |  |
| T3 | 0.52 | (0.15 | -1.85) |  |  |
|  |  |  |  |  |  |
| **IgM N OC43** |  |  |  |  |  |
| T1 | 1.00 |  |  | 0.470 |  |
| T2 | 1.10 | (0.34 | -3.58) |  |  |
| T3 | 0.53 | (0.14 | -2.01) |  |  |
|  |  |  |  |  |  |
| continuous^c^ | 0.38 | (0.08 | -1.90) | 0.240 |  |
|  |  |  |  |  |  |

* This Table shows results for the 18 immunoglobulins least related to COVID-19; results for the other 6 immunoglobulins are shown in Table 8. The odds ratios quantify the magnitude of the associations between the exposures and COVID-19 in the 20 individuals with COVID-19 and the 134 individuals without the disease.

T1 to T3: tertiles.

^a^ Unless otherwise specified, odds ratios were adjusted for age, smoking, and educational level.

^b^ Unless otherwise specified, *p*‑value derived from Wald’s test.

^c^ Odds ratio for each increase of 10 times in the level of the isotype-antigen pair.

^d^ Multivariate analogue of Mantel’s extension test for linear trend.

**Supplementary Table 5.** Effect of total immunoglobulins measured in 2016-17

on the risk of COVID-19 in 2020-21 (N=154)*

| **Immunoglobulin** | **OR^a^** | **(95% CI)** | | **P^b^** |  |  |
| --- | --- | --- | --- | --- | --- | --- |
|  |  |  |  |  |  |  |
| **Ig G1** |  |  |  |  |  |  |
| T1 | 1.00 |  |  | 0.102 |  |  |
| T2 | 0.18 | (0.04 | -0.88) |  |  |  |
| T3 | 0.59 | (0.20 | -1.72) |  |  |  |
|  |  |  |  |  |  |  |
| T1 | 1.00 |  |  | 0.072 |  |  |
| T2+T3 | 0.40 | (0.15 | -1.08) |  |  |  |
|  |  |  |  |  |  |  |
| continuous^c^ | 0.33 | (0.02 | -5.76) | 0.444 |  |  |
|  |  |  |  |  |  |  |
| **Ig G2** |  |  |  |  |  |  |
| T1 | 1.00 |  |  | 0.962 |  |  |
| T2 | 0.97 | (0.28 | -3.39) |  |  |  |
| T3 | 0.85 | (0.25 | -2.92) |  |  |  |
|  |  |  |  |  |  |  |
| continuous^c^ | 0.08 | (0.00 | -3.95) | 0.204 |  |  |
|  |  |  |  |  |  |  |
| **Ig G3** |  |  |  |  |  |  |
| T1 | 1.00 |  |  | 0.156 |  |  |
| T2 | 0.37 | (0.10 | -1.36) |  |  |  |
| T3 | 0.38 | (0.12 | -1.19) |  |  |  |
|  |  |  |  |  |  |  |
| T1 | 1.00 |  |  | 0.054 |  |  |
| T2+T3 | 0.37 | (0.14 | -1.02) |  |  |  |
|  |  |  |  |  |  |  |
| continuous^c^ | 0.04 | (0.00 | -1.27) | 0.068 |  |  |
|  |  |  |  |  |  |  |
| **Ig G4** |  |  |  |  |  |  |
| T1 | 1.00 |  |  | 0.730 |  |  |
| T2 | 1.60 | (0.45 | -5.71) | 0.508 | ^d^ |  |
| T3 | 1.55 | (0.44 | -5.50) |  |  |  |
|  |  |  |  |  |  |  |
| continuous^c^ | 1.71 | (0.42 | -6.88) | 0.451 |  |  |
|  |  |  |  |  |  |  |
| **Sum IgGs** |  |  |  |  |  |  |
| T1 | 1.00 |  |  | 0.451 |  |  |
| T2 | 0.48 | (0.13 | -1.77) |  |  |  |
| T3 | 0.56 | (0.18 | -1.73) |  |  |  |
|  |  |  |  |  |  |  |
| T1 | 1.00 |  |  | 0.213 |  |  |
| T2+T3 | 0.53 | (0.19 | -1.45) |  |  |  |
|  |  |  |  |  |  |  |
| continuous^c^ | 0.09 | (0.00 | -4.26) | 0.219 |  |  |
|  |  |  |  |  |  |  |
| **Ig E** |  |  |  |  |  | * The odds ratios quantify the magnitude of the associations between the immunoglobin isotypes and subclasses concentrations (µg/mL), and COVID-19 in the 20 individuals with COVID-19 and the 134 individuals without the disease.  T1 to T3: tertiles.  ^a^ Odds ratios were adjusted for age, smoking, and educational level.  ^b^ Unless otherwise specified, *p*‑value derived from Wald’s test.  ^c^ Odds ratio for each increase of 10 times in the level of the immunoglobin isotypes and subclasses.  ^d^ Multivariate analogue of Mantel’s extension test for linear trend. |
| T1 | 1.00 |  |  | 0.606 |  |  |
| T2 | 1.56 | (0.42 | -5.83) | 0.324 | ^d^ |  |
| T3 | 1.88 | (0.54 | -6.54) |  |  |  |
|  |  |  |  |  |  |  |
| continuous^c^ | 2.87 | (0.43 | -18.92) | 0.274 |  |  |
|  |  |  |  |  |  |  |
| **Ig A** |  |  |  |  |  |  |
| T1 | 1.00 |  |  | 0.246 |  |  |
| T2 | 0.88 | (0.29 | -2.62) | 0.115 | ^d^ |  |
| T3 | 0.31 | (0.07 | -1.30) |  |  |  |
|  |  |  |  |  |  |  |
| T1+T2 | 1.00 |  |  | 0.097 |  |  |
| T3 | 0.33 | (0.09 | -1.22) |  |  |  |
|  |  |  |  |  |  |  |
|  |  |  |  |  |  |  |
| continuous^c^ | 0.03 | (0.00 | -0.73) | 0.032 |  |  |
|  |  |  |  |  |  |  |
| **Ig M** |  |  |  |  |  |  |
| T1 | 1.00 |  |  | 0.530 |  |  |
| T2 | 0.79 | (0.22 | -2.78) | 0.264 | ^d^ |  |
| T3 | 0.48 | (0.13 | -1.76) |  |  |  |
|  |  |  |  |  |  |  |
| continuous^c^ | 0.30 | (0.02 | -4.55) | 0.389 |  |  |
|  |  |  |  |  |  |  |

**Supplementary Table 6.** Influence of mixtures of total immunoglobins

on the risk of COVID-19 (N = 154)

| **Model** | | **OR^a^** | **(95% CI)** | | **P^b^** |
| --- | --- | --- | --- | --- | --- |
| **1** | **IgG1** |  |  |  |  |
|  | T1 | 1.00 |  |  | 0.105 |
|  | T2+T3 | 0.43 | (0.15 | -1.21) |  |
|  |  |  |  |  |  |
|  | **IgG3** |  |  |  |  |
|  | T1 | 1.00 |  |  | 0.078 |
|  | T2+T3 | 0.40 | (0.14 | -1.12) |  |
|  |  |  |  |  |  |
| **2** | **IgG3** |  |  |  |  |
|  | T1 | 1.00 |  |  | 0.081 |
|  | T2+T3 | 0.41 | (0.14 | -1.13) |  |
|  |  |  |  |  |  |
|  | **IgA** |  |  |  |  |
|  | T1+T2 | 1.00 |  |  | 0.135 |
|  | T3 | 0.36 | (0.08 | -1.22) |  |
|  |  |  |  |  |  |

^a^ Odds ratios of the total immunoglobulins are mutually adjusted for,

and further adjusted by age, education and smoking (all three confounders p<0.25).

^b^ Wald’s test (two-tailed).
